# JMJD3-mediated HADH demethylation orchestrates hepatocyte maturation through fatty acid β-oxidation-dependent histone acetylation

**DOI:** 10.1038/s12276-026-01816-x

**Published:** 2026-09-01

**Authors:** Haibin Wu, Sen Chen, Huanhuan Shan, Wenjiao Yan, Changlu Qin, Shoupei Liu, Xiangting Cao, Jinzhi Du, Yongjian Zhou, Yuyou Duan

**Affiliations:** 1 Department of Gastroenterology and Hepatology, Guangzhou Digestive Disease Center, The Second Affiliated Hospital, School of Medicine, South China University of Technology, Guangzhou, China; 2 Laboratory of Stem Cells and Translational Medicine, Center for Medical Research on Innovation and Translation, Institute of Clinical Medicine, The Second Affiliated Hospital, School of Medicine, South China University of Technology, Guangzhou, China; 3 Laboratory of Stem Cells and Translational Medicine, Institute for Life Science, School of Medicine, South China University of Technology, Guangzhou, China; 4 School of Medicine, South China University of Technology, Guangzhou, China; 5 National Engineering Research Center for Tissue Restoration and Reconstruction, South China University of Technology, Guangzhou, China; 6 The Innovation Centre of Ministry of Education for Development and Diseases, The Second Affiliated Hospital, School of Medicine, South China University of Technology, Guangzhou, China

**Keywords:** Embryonic stem cells, Stem-cell differentiation

## Abstract

Functionally mature hepatocyte generation requires coordinated genetic, metabolic, and epigenetic regulation. Although transcriptional regulation governing hepatocyte differentiation has been extensively studied, how metabolic remodeling interacts with epigenetic regulation during hepatocyte maturation remains poorly understood. Using a human embryonic stem cell-derived hepatocyte organoid differentiation model, we identified JMJD3 as a critical regulator of hepatocyte maturation through metabolic–epigenetic coupling. Mechanistically, JMJD3 activated the fatty acid β-oxidation (FAO) enzyme HADH by removing repressive H3K27me3 marks at its locus, thereby promoting FAO activity during hepatocyte maturation. Enhanced FAO increased acetyl-CoA production, which subsequently promoted EP300-mediated H3K27ac deposition at hepatocyte maturation-associated genes. Functional inhibition of JMJD3, HADH, or FAO impaired hepatocyte maturation, resulting in lipid droplet accumulation and reduced expression of hepatocyte maturation markers. Conversely, restoration of acetyl-CoA partially rescued these maturation defects, supporting a functional link between FAO-dependent metabolism and histone acetylation. Collectively, our findings establish a JMJD3–HADH–FAO–acetyl–CoA regulatory axis that coordinates metabolic remodeling and epigenetic regulation during hepatocyte maturation.

## JMJD3 links metabolism and epigenetics in hepatocyte maturation

Primary human hepatocytes are the benchmark for liver research; however, their use is limited by donor variability and functional decline in culture. This study explores the role of JMJD3, an epigenetic regulator, in human hepatocyte maturation using human embryonic stem cell-derived organoids. Researchers used pharmacological and genetic tools to modulate fatty acid oxidation (FAO) and JMJD3 expression, revealing that JMJD3 links FAO to histone modification, crucial for maturation. They found that JMJD3 knockdown disrupted lipid metabolism and hepatocyte function, highlighting its role in regulating maturation through FAO-dependent acetyl-CoA production. This acetyl-CoA is vital for histone acetylation, promoting gene expression necessary for maturation. The study suggests JMJD3 as a potential target for treating metabolic liver diseases and underscores the need for further research into its therapeutic applications. This summary was initially drafted using artificial intelligence, then revised and fact-checked by the author.

## Introduction

Primary human hepatocytes (PHHs) are considered the gold standard in vitro model for liver-related disease research, drug development, and cell therapy^1^. However, their broad application is hindered by limited sources, variability between donors, and function loss under conventional culture conditions. To address these challenges, alternative cell sources have been explored, including transdifferentiated hepatocytes^2^, PHH-derived hepatic progenitor cells^3^, mesenchymal stem-cell-derived hepatocytes^4^, and pluripotent stem cell (PSC)-differentiated hepatocytes^5^. Nevertheless, owing to incomplete understanding of regulation networks governing hepatocyte maturation, it is still a challenge to generate or maintain functional mature hepatocyte equivalent to PHHs, which critically hampers the translational applications of these differentiated hepatocytes.

Important advances have been made in delineating transcriptional regulators governing hepatocyte differentiation and functional maintenance. Core transcription factors including HNF4α and CEBPα orchestrate hepatocyte-specific transcriptional programs essential for the maturation^6^. Beyond transcriptional control, emerging evidence underscores the indispensable roles of epigenetic remodeling and metabolic reprogramming during hepatocyte development^7^. In murine studies, histone modifications have been demonstrated to critically regulate hepatoblast development^8,9^, whereas metabolic remodeling dictates cell fate decisions during the development^10,11^. However, interspecies differences substantially hamper direct translation of these findings to human biology^12–15^. The emergence of human organoid culture systems has provided a transformative experimental platform that mitigates species-specific limitations, enabling modeling of drug responses, developmental processes, and disease pathogenesis with enhanced human relevance in vitro^16^. Notably, human pluripotent stem cell (hPSC)-derived hepatocyte organoids recapitulate in vivo hepatocyte characteristics, including the polarity, phase I/II enzymatic activity, and transcriptomic profiles similar to primary hepatocytes^17–19^. This functional and structural fidelity establishes organoids as a powerful tool for dissecting human hepatocyte development mechanisms.

Metabolic processes are reported as a critical driver of the organogenesis. Compared with venous endothelial cells, differentiated lymphatic vessels exhibited increased fatty acid β-oxidation (FAO) activity, which is important for lymphatic development^20^. In addition, the metabolic transition between fatty acid synthesis and FAO is indispensable for definitive endoderm differentiation from hPSCs^21^. Although analogous metabolic transition occurs during hepatocyte differentiation^22^, the contributions of lipid metabolism in hepatocyte maturation are not yet understood.

Epigenetic regulatory factor JMJD3 (*KDM6B*) together with UTX (*KDM6A*) belongs to KDM6 family and epigenetically activates genes by demethylating H3K27me3 marks^23^. JMJD3 has a crucial role in regulating hematopoietic stem cell self-renewal^24^ and neurogenesis from hPSCs^25^, indicating its fundamental role in fate determination. Despite recognized importance of epigenetic remodeling in hepatic maturation^8^, the mechanism of JMJD3 regulation in hepatocyte maturation remains uncharacterized.

In this study, using human embryonic stem cell (hESC)-derived hepatoblast organoids (HB-orgs) to differentiate into mature polarized hepatocyte organoids (P-heporgs)^19^, we established a stage-specific differentiation model to delineate fatty acid metabolism dynamics. Furthermore, we identified JMJD3 as a critical mediator linking FAO to histone modification and transcriptional activation of maturation-associated genes. To dissect the role of FAO and JMJD3 in hepatocyte maturation, pharmacological modulators and doxycycline (Dox)-inducible short hairpin (shRNA) knockdown technology were employed to selectively inhibit FAO activity and JMJD3 expression. Cut&Tag chromatin profiling further mapped JMJD3-dependent histone modification loci at maturation-related genes. Collectively, our findings uncover unprecedented links connecting JMJD3-mediated epigenetic regulation with FAO metabolism and human hepatocyte maturation, providing new insights into hepatocyte development.

## Materials and methods

### Cell culture and differentiation

The hESC line H9 cells were obtained from WiCell Research Institute (Madison, WI, USA) under Materials Transfer Agreements (Nos 19-W0512, 24-W0162, and 24-W0163). H9 cells were cultured on mouse embryonic fibroblast feeder layers in Dulbecco's modified Eagle's medium/F12 medium (Gibco, C11330500BT), which contains 20% knockout serum replacement (Gibco, 10828028), 1% non-essential amino acids (Gibco, 11140050), 0.1 mM 2-mercaptoethanol (Sigma, M3148), 1% GlutaMax I (Gibco, 35050061), and 10 ng/ml bFGF (PeproTech, 100-18B). H9 cells were maintained in a humidified incubator at 37 °C and 5% CO_2_. The culture medium was changed daily, and H9 colonies were dissociated into small clumps by collagenase IV (Gibco, C0130) and transferred onto a fresh feeder layer every 5–6 days.

The differentiation and culture of HB-orgs and P-heporgs were performed as described previously^19^. Briefly, HB-orgs were cultured in HB-expansion medium that consisted of IMDM (Gibco, C12440500BT), 10% fetal bovine serum (ExCell Bio, FND500), 1% ITSE (Neobioscience, 00-101-10), 1% NEAA, 1% GlutaMax I, 0.3 mM 1-thioglycerol (Sigma, M6145), 10 mM nicotinamide (Sigma, N0636), 5 μM CHIR99021 (MedChemExpress, HY-10182), 10 μM SB431542 (MedChemExpress, HY-10431), 10 μM Forskolin (MedChemExpress, HY-15371), 20 ng/ml BMP4 (PeproTech, 120-05), 20 ng/ml FGF4 (PeproTech, 100-31), 20 ng/ml EGF (PeproTech, AF-100-15), and 10 μM Y27632 (MedChemExpress, HY-10071) for 1 day, supplemented with dispersing 5% growth factor-reduced Matrigel (Corning, 354230), and cultured in ultra-low attachment 6-well plates (Corning, 3471). For culture of P-heporgs, HB-orgs were directly transferred to hepatocyte culture medium (HCM, Lonza, CC-3199) supplemented with SingleQuots (Lonza, CC-4182) minus EGF, supplemented with 1% B27 (Gibco, 17504044), 100 nM dexamethasone (Sigma, D4902), 20 ng/ml FGF4, 20 ng/ml HGF (PeproTech, 100-39), and 40 ng/ml Oncostatin M (PeproTech, 300-10); 5% ice-cold growth factor-reduced Matrigel was added into the medium and HB-orgs were transferred. The medium was changed every day.

The differentiation of hepatocyte-like cells (HLCs) under 2D culture conditions was performed, as described previously^5^. Briefly, hESCs were sequentially induced toward hepatic lineage. For definitive endoderm induction, cells were cultured in RPMI-1640 medium supplemented with 100 ng/ml Activin A (PeproTech, 120-14) and 25 ng/ml Wnt3a (PeproTech, 315-20) for 2 days, followed by additional 5 days in fresh RPMI-1640 medium containing 100 ng/ml Activin A, 0.5 mM sodium butyrate, and 1% B27 supplement. Subsequently, hepatoblast differentiation was performed for 14 days in hepatocyte differentiation medium consisting of IMDM supplemented with 20% FBS, 0.3 mM 1-thioglycerol, 0.5% dimethyl sulfoxide, 100 nM dexamethasone, 20 ng/ml HGF (PeproTech, 294-HG), 20 ng/ml FGF4, 10 ng/ml BMP2 (PeproTech, 120-02), and 10 ng/ml BMP4, and the medium was changed every 2 days. For hepatocyte maturation, cells were further cultured in HCM as described earlier for 8 days to generate HLCs.

### Animal experiments

All animal procedures were approved by Research Ethics Committee of School of Medicine at South China University of Technology (Ethical Approval No. 2019073). The livers at E12.5 from BALB/C mice (Zhiyuan Biomedical Technology Co., Ltd) were cultured on Millicell Cell Culture Insert (Merck, PICM0RG50) in 6-well plates, containing 2 ml of RPMI-1640 medium (Gibco, C11875500BT) supplemented with 10% fetal bovine serum, 50 ng/ml EGF, 1×ITS-X (ThermoFisher, 51500056), and antibiotics. The cultures were treated for 2 days with 10 μM GSK-J4 (MedChemExpress, HY-15648B) or acetate (Sigma, S5636) or dimethyl sulfoxide (DEWEIBIO, 0219605580) as a control. The medium was changed every day.

### Plasmid construction, lentiviral packaging, and cell infection

shRNAs targeting human UTX and JMJD3 were cloned into Tet-pLKO-Puro vector (Honorgene, HG-VRH1736), and shRNA targeting human HADH was cloned into Tet-pLKO-ZsGreen1-Puro vector (shRNA sequences were listed in Supplementary Table 3). The full-length human JMJD3 cDNA was cloned into pTetOn vector. A demethylase-deficient JMJD3 mutant (H1388A), generated by mutating histidine 1388 to alanine via homologous recombination (Yeasen, Cat. no. 10922ES20), was also cloned into pTetOn vector. All constructs were confirmed by Sanger sequencing. Lentiviral particles were produced as described previously^26^. To transduce lentivirus particles into HB-orgs, single cells from HB-orgs were seeded into HB-expansion medium supplemented with virus stock and 2 μg/ml polybrene (Sigma, TR-1003). The medium was replaced after 24 h incubation. When passaged, 2 μg/ml puromycin was added for the selection of transduced cells. When HB-orgs were differentiated into P-heporgs, Dox was added to the medium at a final concentration of 2 μg/ml to induce the expression of shRNA or JMJD3 on hepatocyte maturation stage.

### Quantitative reverse transcription–PCR

Total RNAs were extracted using RNAiso Plus kit (Takara, 9109) according to the manufacturer’s instructions. One microgram of RNA was reverse-transcribed into cDNA using HiScript III RT SuperMix for qPCR (Vazyme, R323-01). Quantitative reverse transcription–PCR (qRT–PCR) was performed using ChamQ Universal SYBR qPCR Master Mix (Vazyme, Q711-03) and 96-well qPCR plate (MIKX, MK1009) on QuantStudio 1 Real-Time PCR system (ABI, Thermo, USA). The cycle threshold (CT) values for each sample were normalized to the expression of the housekeeping gene GAPDH. Relative gene expression levels were calculated using the 2^−ΔΔCT^ method. The primer sequences used for qRT–PCR were listed in Supplementary Table 1.

### Immunofluorescence staining

Organoids were collected at designated time points, washed with PBS, and fixed overnight at 4 °C in 4% paraformaldehyde. Following the fixation, the organoids were permeabilized with 0.5% Triton X-100 for 20 min and blocked with goat or donkey serum for 60 min. After each step, organoids were washed three times with PBS. Organoids were then incubated with primary antibodies diluted in PBS overnight at 4 °C, followed by the incubation with secondary antibodies in PBS for 1 h at room temperature in the dark. Nuclei were counterstained with 4′,6-diamidino-2-phenylindole (DAPI) (Beyotime Biotechnology, C1006) for 5 min. Immunostaining images were captured using a single-photon confocal microscope (LSM 900, Zeiss, Germany). The antibodies used in this study were listed in Supplementary Table 2.

### Western blot analysis

Cells were lysed on ice using RIPA lysis buffer (Solarbio, R0020) supplemented with phenylmethylsulfonyl fluoride (Beyotime Biotechnology, ST505) and protease inhibitor (Beyotime Biotechnology, P1005). Protein concentrations were determined using bicinchoninic acid Protein Assay Kit (Biosharp, BL521A), following manufacturer’s instructions. Western blotting (WB) was performed using standard procedures. The antibodies used in this study were listed in Supplementary Table 2.

### ChIP-qPCR

By using chromatin immunoprecipitation (ChIP) assay kit (Beyotime Biotechnology, P2078), the supernatant of sonicated organoids was co-immunoprecipitated with anti-H3K27me3 (CST, 9733T) or anti-JMJD3 antibodies (Abcepta, AP1022A) to assess the binding with HADH, respectively. Rabbit IgG was used as negative control. The enrichment of target gene fragment in DNA precipitate was analyzed by quantitative real-time PCR, and the primers for target genes were listed in Supplementary Table 1. The fold enrichments of target genes between ChIP-DNA and input-DNA were determined by ΔCt.

### Analysis for liver function

To analyze the secretion levels of albumin (ALB), medium was collected at indicated time points and analyzed by using Human Albumin ELISA Quantitation Kit (Bethyl, E88-129) following manufacturer’s instructions. To analyze the production of urea, 10 mM ammonium chloride (Sigma, A9434) was added into the culture medium and incubated for 24 h, and then the medium was collected and Urea Assay Kit (Solarbio, BC1535) was used for quantification according the manufacturer’s protocols.

### Culturing organoids in high-fat environment

P-heporgs infected with LV-Ctrl/JMJD3-shRNA were treated with a low concentration of free fatty acid (FFA) (100 μM), composed with oleic acid (Sigma, O1383): palmitic acid (Sigma, P0500) = 2:1 for 2 days. P-heporgs infected with LV-vector/JMJD3-mutant/overexpression were treated with a high concentration of FFA (300 μM) for 2 days. Organoids were collected and analyzed on indicated time points.

### Lipid droplet staining

Organoids were washed with PBS and fixed with 4% paraformaldehyde for 20 min at room temperature. After washing with PBS three times, organoids were stained with BODIPY 493/503 (GLPBIO, GC42959) or Nile red (Beyotime Biotechnology, C2051S) solution for 30 min. After washing with PBS three times, nuclei were counterstained with DAPI for 5 min. The fluorescent images were captured using a single-photon confocal microscope. The relative lipid area was quantitative by using ImageJ software.

### Triglyceride assay

To detect the concentrations of intracellular triglyceride (TG), organoids were collected and analyzed with a Triglyceride Assay Kit (Njjcbio, A110-1), according to the manufacturer’s protocols.

### Acetyl-CoA and FAO level measurement

To measure acetyl-CoA concentration and FAO levels, organoids were collected, washed with PBS, and lysed on ice using sonication. The cell lysate was measured with Acetyl-CoA assay kit (MEIMIAN, MM-14788H2) or Fatty Acid Oxidation assay kit (Elabscience, E-BC-K784-M), following manufacturer’s instructions. Cell mass was normalized to cellular protein concentration by using Protein Assay Kit.

### RNA-seq analysis

Total RNAs of organoids were extracted using RNAiso Plus kit according to the manufacturer’s instructions. The cDNA libraries for RNA sequencing were generated using NEBNext® Ultra™ RNA Library Prep Kit for Illumina (NEB, E7530). Sequencing was performed by Novogene (Beijing, China) on an Illumina HiSeq X-Tensequencer with 150 bp paired-end sequencing reaction. Differentially expressed genes (DEGs) were analyzed by DESeq2 using counts. Genes with *P*-value ≤ 0.05 and fold change ≥ 2 were identified as DEGs. Original data were uploaded to the Gene Expression Omnibus database (accession number: GSE295093).

### Cut&Tag

Organoids treated with different shRNAs were trypsinized into single cells with TrypLE (Gibco, 12604021) and followed by manufacturer’s instructions of Hyperactive Universal CUT&Tag Assay Kit for Illumina (Vazyme, TD903) to obtain DNA fragments bounded by primary antibodies H3K27me3 (CST, 9733T) or H3K27ac (CST, 8173T). Subsequently, DNA library preparation and amplification were conducted by TruePrepR Index Kit V2 for Illumina (Vazyme, TD202). The resulting PCR products were purified by VAHTS DNA Clean Beads (Vazyme, N411). Sequencing was performed by Novogene (Beijing, China) on a NovaSeq X Plus. Original data were uploaded to Gene Expression Omnibus database (accession number: GSE297175). Human hepatocyte maturation-related gene data were downloaded from Human Protein Atlas database.

### Statistical analysis

Sample sizes were indicated on the corresponding graph or figure legend; otherwise, *n* = 3, which represented the number of biological replicates that were analyzed in each experimental group. Data were expressed as mean ± standard deviation (SD) or standard error of mean (SEM). Statistical analysis was performed using SPSS. The unpaired two-tailed Student’s *t* test and one-way analysis of variance (Tukey correction for multiple comparisons) were used to evaluate statistical significance. Differences were considered statistically significant at *P*-value < 0.05.

## Results

### H3K27me3 demethylases were required for hepatocyte maturation

**Fig. 1:**
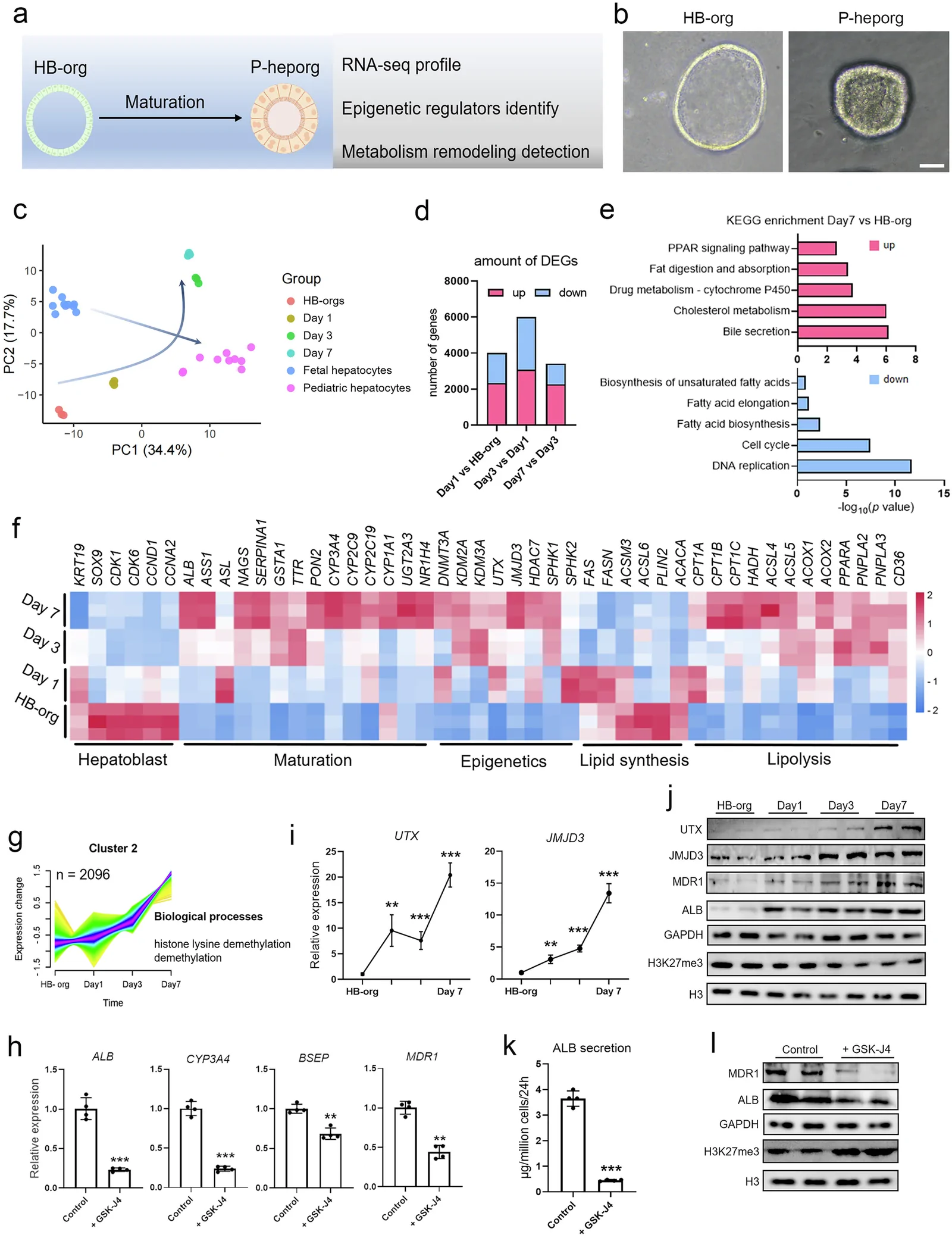
H3K27me3 demethylases were required for hepatocyte maturation. **a** Schematic illustration of the experimental design. **b** The representative images of hepatoblast organoid (HB-org) and polarized hepatocyte organoid (P-heporg). Scale bar, 50 μm. **c** Principal component analysis plot including HB-orgs, differentiated hepatocyte organoids, human fetal liver, and pediatric hepatocyte samples (GSE111845) demonstrated progressive maturation trajectory. **d** Cumulative plots of the amount of differentially expressed genes (DEGs) per time point post the differentiation (|log 2 FC | > 0.5, *P*-adj < 0.05). **e** KEGG enrichment analysis on the upregulated and downregulated DEGs identified at day 7 versus HB-orgs. **f** Heatmap for genes related to hepatoblast, maturation, epigenetics, lipid synthesis, and lipolysis. **g** Partial results of hierarchical clustering and Gene Ontology analysis of all DEGs (the complete results were showed in Supplementary Fig. 1a). **h** The expressions of maturation related genes were analyzed by quantitative reverse transcription–PCR (qRT–PCR) in P-heporgs with or without GSK-J4 treatment when compared with those in control. **i** Dynamic gene expressions of UTX and JMJD3 were analyzed by qRT–PCR when compared with those in HB-orgs. **j** The expressions of indicated proteins at different time points were analyzed by western blotting. **k** The secretion of ALB in P-heporgs with or without GSK-J4 treatment when compared with those in control. **l** The expression of indicated proteins in P-heporgs with or without GSK-J4 treatment was analyzed by western blotting. Results were presented as mean ± SD. **P < 0.01, ***P < 0.001. ALB, albumin; KEGG, Kyoto Encyclopedia of Genes and Genomes; RNA-seq, RNA sequencing.

Our prior work validated that differentiated HB-orgs and P-heporgs recapitulated the phenotypic and functional features of native hepatoblasts and PHHs, respectively^19^. To further identify key regulators of hepatocyte maturation, we performed RNA sequencing (RNA-seq) to profile transcriptional dynamics of HB-orgs and P-heporgs at distinct differentiation stages (Fig. 1a,b and Supplementary Fig. 1a). To contextualize the developmental identity of our organoids, we incorporated publicly available RNA-seq datasets from human fetal liver and pediatric hepatocytes (GSE111845), representing distinct stages of postnatal hepatocyte maturation, for comparative transcriptomic analysis. Principal component analysis revealed a clear developmental trajectory across samples (Fig. 1c). HB-orgs and early differentiation samples (day 1) clustered closely with fetal liver samples, whereas more differentiated organoids (day 3 and day 7) shifted toward pediatric hepatocytes, indicating progressive maturation toward a more advanced hepatocyte state. These findings demonstrate that the transcriptional progression of our organoids recapitulates developmental transitions observed during human liver maturation in vivo. Consistently, more than 6,000 genes exhibited significant expression changes during the differentiation, with the most pronounced transcriptional remodeling occurring within the initial 72 h (Fig. 1d). KEGG pathway analysis of P-heporgs on day 7 versus HB-orgs demonstrated that upregulated genes were enriched in mature hepatocyte functions, including cytochrome P450-mediated drug metabolism, cholesterol homeostasis, and bile acid secretion (Fig. 1e). Conversely, downregulated genes were associated with proliferative pathways such as cell cycle regulation and DNA replication (Fig. 1e). Furthermore, heatmap analysis documented sequential silencing of hepatic progenitor markers alongside the activation of maturation-associated genes (Fig. 1f), validating the successful differentiation of functional hepatocyte organoids.

DEGs were classified into nine distinct clusters based on temporal expression patterns (Supplementary Fig. 1a). Clusters 6 and 7, marked by progressive downregulation during the differentiation, were predominantly enriched in cell cycle-related biological processes (Supplementary Fig. 1a). Conversely, genes in cluster 2 exhibiting sustained upregulation demonstrated robust enrichment in epigenetic regulatory pathways, notably histone demethylation (Fig. 1g), implicating epigenetic remodeling as a critical driver of hepatocyte maturation. To validate these findings, pharmacological inhibitors targeting highly expressed epigenetic regulators were used, and we finally identified GSK-J4, a dual UTX/JMJD3 inhibitor, as a selective suppressor of hepatocyte maturation gene (Fig. 1h). By contrast, inhibitors targeting DNMT3A, HDAC7, or SPHK1/2 exhibited no discernible effects (Supplementary Fig. 1b). Consistent with these observations, WB and qRT–PCR analyses revealed a temporal correlation between the upregulation of UTX/JMJD3 and the induction of maturation markers ALB and multidrug resistance protein 1 (MDR1), paralleling a global decline in H3K27me3 levels during the differentiation (Fig. 1i,j). Functional validation assays further demonstrated that GSK-J4 treatment not only suppressed ALB secretion and ALB/MDR1 protein expression but also elevated H3K27me3 accumulation (Fig. 1k,l). Collectively, these results revealed that UTX/JMJD3 may have a critical role in the differentiation and maturation of hepatocyte organoids.

### JMJD3 was required for hepatocyte maturation

**Fig. 2:**
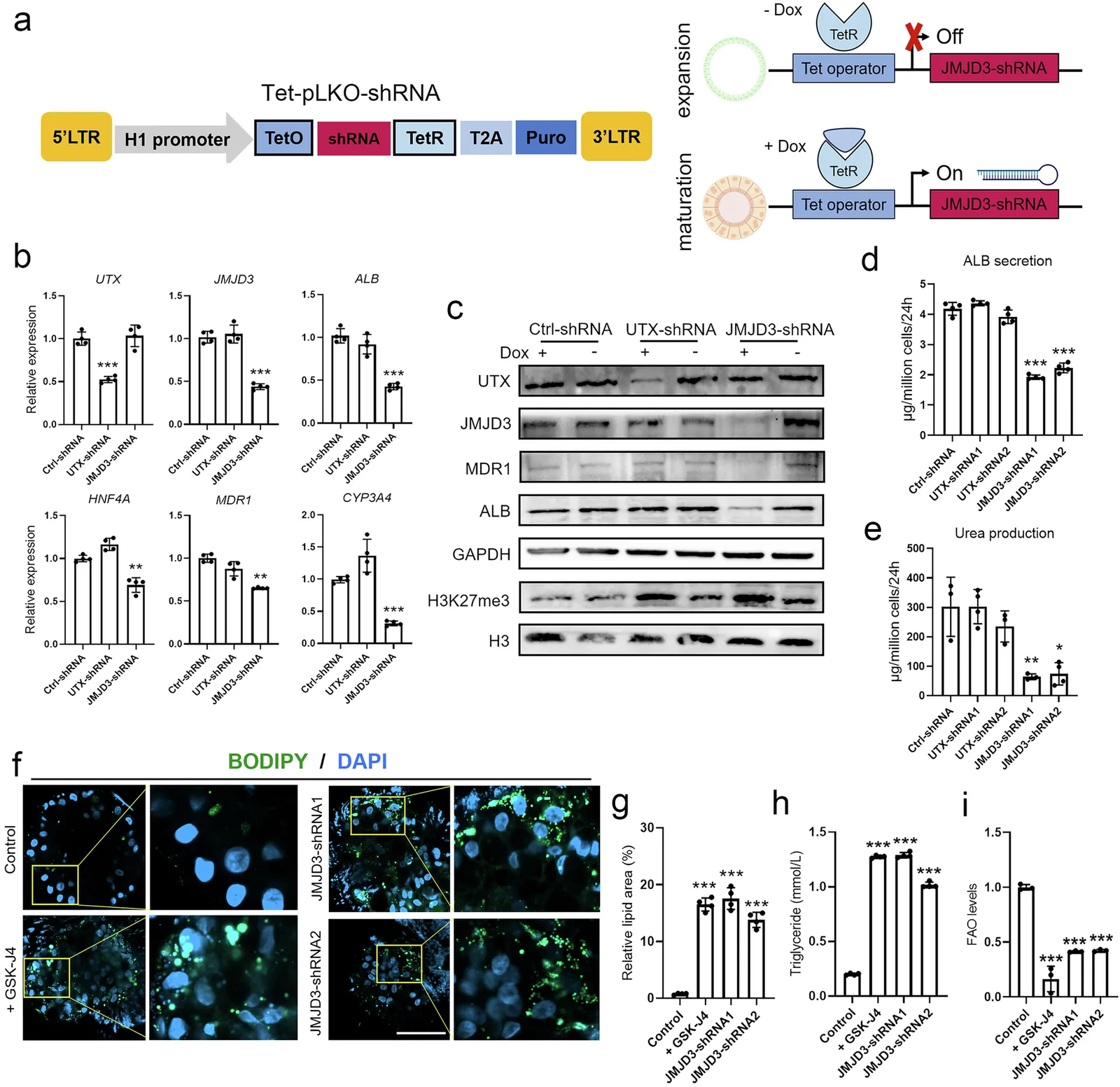
JMJD3 but not UTX was required for hepatocyte maturation. **a** Schematic illustration of doxycycline (Dox)-induced short hairpin RNAs (shRNAs). **b** The expressions of maturation-related genes were analyzed by quantitative reverse transcription–PCR (qRT–PCR) in polarized hepatocyte organoids (P-heporgs) treated with UTX/JMJD3-shRNAs when compared with ctrl-shRNA group. **c** The expressions of indicated proteins in P-heporgs treated with or without UTX or JMJD3-shRNA and Dox. The analysis of the secretion of albumin (ALB) (part **d**) and the production of urea (part **e**) when compared with ctrl-shRNA group. **f** The representative fluorescent images of BODIPY in P-heporgs treated with JMJD3-shRNA or GSK-J4. Nuclei were stained with DAPI. Scale bar, 100 μm. The analysis of relative lipid area (part **g**), the content of intracellular triglycerides (part **h**), and the levels of fatty acid β-oxidation (FAO) (part **i**) in P-heporgs treated with JMJD3-shRNA or GSK-J4 when compared with control. Results were presented as mean ± SD. *P < 0.05, **P < 0.01, ***P < 0.001. DAPI, 4′,6-diamidino-2-phenylindole.

To investigate whether UTX and JMJD3 regulated hepatocyte maturation independently or synergistically, we constructed Dox-inducible shRNA-expressing lentiviral vector targeting UTX or JMJD3 (Fig. 2a). During the differentiation of HB-orgs into P-heporgs, Dox was administered to trigger specific shRNA expression for gene knockdown. Intriguingly, only JMJD3 knockdown significantly reduced the expression of hepatocyte maturation genes (*ALB*, *HNF4*α, *MDR1*, and *CYP3A4*), whereas UTX knockdown showed no substantial effects (Fig. 2b). Furthermore, the expression of *KDM6B* (JMJD3) and the expression of mature hepatocyte genes (*CYP3A4*, *CYP2C9*, *ABCB1*, *SERPINA1*, *HNF4A*, and *HADH*) increased progressively from fetal to pediatric hepatocytes (GSE111845), whereas *KDM6A* (UTX) expression remained unchanged (Supplementary Fig. 2a). This analysis indicated that JMJD3 upregulation during hepatocyte maturation was a physiologically relevant event, whereas the increased expression of UTX observed in organoids likely represented an in vitro-specific change without functional consequence. To further validate these observations, WB and immunofluorescence analyses confirmed that JMJD3 knockdown effectively diminished its protein expression, concurrently suppressing ALB/MDR1 expression and modulating H3K27me3 levels (Fig. 2c and Supplementary Fig. 2b). Functional assessments also revealed that JMJD3-knockdown markedly impaired ALB secretion and urea synthesis capacities of P-heporgs compared with those in the control group (Fig. 2d,e). Collectively, these data demonstrated that JMJD3, but not UTX, had a critical role in regulating hepatocyte maturation, and its dynamic regulation in organoids mirrors transcriptional changes occurring during normal human liver development.

### FAO level affected hepatocyte maturation

We observed that JMJD3 knockdown not only disrupted the expression of hepatocyte maturation-related genes/proteins and impaired hepatocyte functions but also markedly perturbed intracellular lipid metabolism. JMJD3-knockdown organoids exhibited excessive lipid droplet accumulation (Fig. 2f,g) and elevated TG levels (Fig. 2h), accompanied by significantly reduced FAO activity (Fig. 2i). Transcriptomic profiling revealed that HB-orgs displayed strong upregulation of lipogenic genes (*FAS*, *FASN*, and *PLIN2*) and fatty acid biosynthesis pathways (Fig. 1e,f). By contrast, mature P-heporgs showed increased expression of lipolysis genes (*CPT1A*, *CPT1B*, and *HADH*) and enrichment of lipid degradation pathways (Fig. 1e,f), indicating enhanced lipid catabolism capacity in differentiated P-heporgs.

**Fig. 3:**
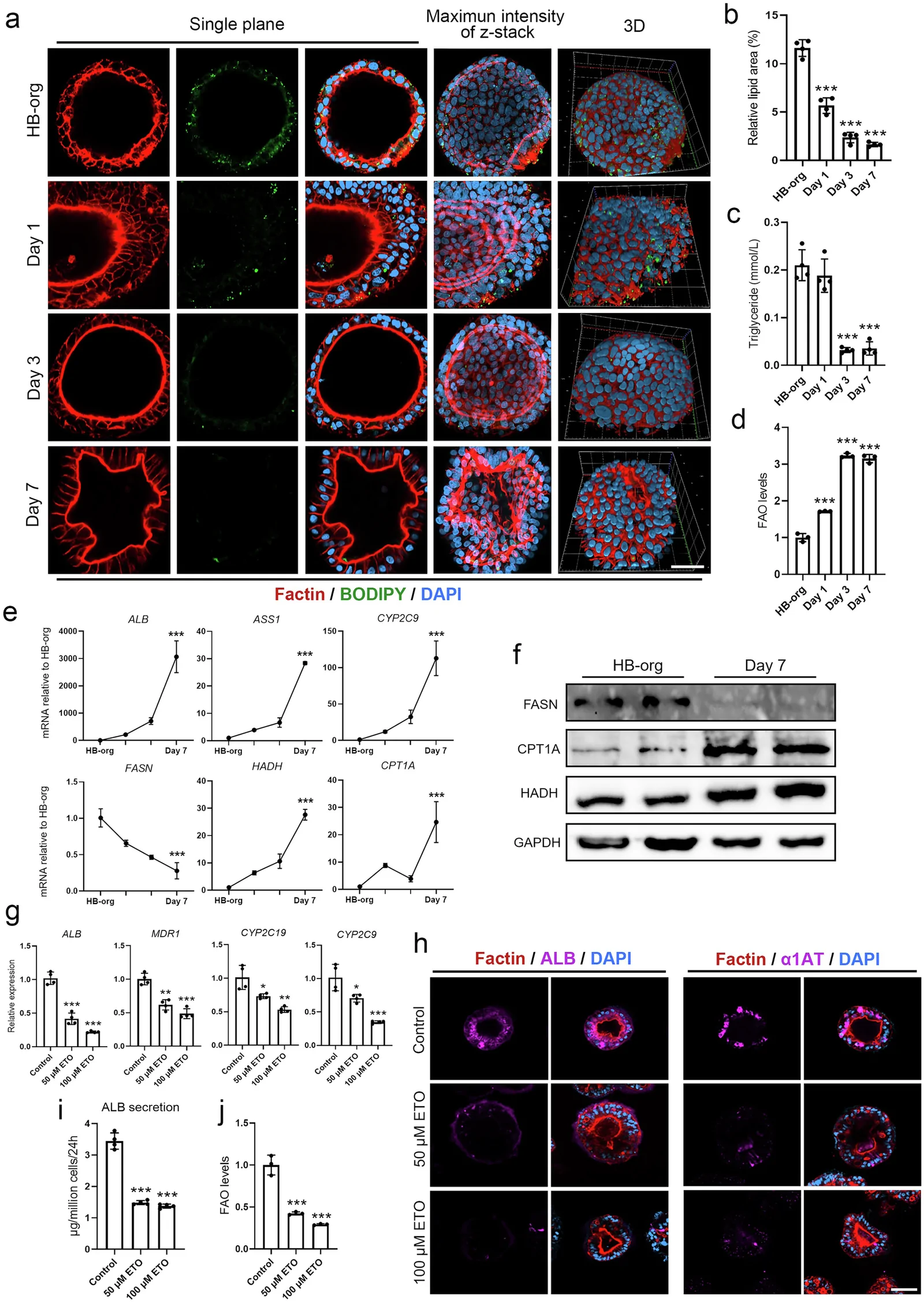
FAO levels affected hepatocyte maturation. **a** Immunostaining for Factin and lipid droplet (BODIPY staining) in polarized hepatocyte organoids (P-heporgs) at different time points post differentiation. Nuclei were stained with DAPI. Scale bar, 50 μm. The analysis of relative lipid area (part **b**), the content of intracellular triglycerides (part **c**), and the levels of fatty acid oxidation (FAO) (part **d**) in P-heporgs at different time points post differentiation when compared with hepatoblast organoids (HB-orgs). **e** Dynamic gene expressions related to the maturation and lipid metabolism were analyzed by quantitative reverse transcription–PCR (qRT–PCR) when compared with HB-orgs. **f** The expressions of indicated proteins in HB-orgs or P-heporgs on day 7 post differentiation. **g** The gene expressions related to the maturation of P-heporgs treated with or without etomoxir (ETO) were analyzed by qRT–PCR. **h** Immunostaining for Factin and albumin (ALB) or α1AT in P-heporgs treated with or without ETO. Nuclei were stained with DAPI. Scale bar, 100 μm. **i** The analysis of ALB secretion in P-heporgs treated with or without ETO. **j** The analysis of FAO levels in P-heporgs treated with or without ETO when compared with control. Results were presented as mean ± SD. *P < 0.05, **P < 0.01, ***P < 0.001. DAPI, 4′,6-diamidino-2-phenylindole.

To validate this metabolic switch, we quantified lipid dynamics during P-heporgs maturation. The results showed progressive decreases in lipid droplet contents (Fig. 3a,b) and TG levels (Fig. 3c), paralleled by corresponding increases in FAO activity (Fig. 3d). qRT–PCR analysis confirmed temporal upregulation of maturation genes (*ALB*, *ASS1*, and *CYP2C9*) alongside declining *FASN* and rising *CPT1A* and *HADH* expression (Fig. 3e), with WB validation at the protein levels (Fig. 3f). Given that FAO is the predominant catabolic pathway for FFAs in hepatocytes, we assessed its functional relevance to hepatocyte maturation using FAO-specific inhibitor etomoxir (ETO). ETO treatment induced dose-dependent lipid droplet accumulation (Supplementary Fig. 3a,b) while suppressing the expression of maturation-related genes (*ALB*, *MDR1*, *CYP2C19*, and *CYP2C9*) (Fig. 3g), the secretion and expression of ALB and α1AT (Fig. 3h,i) and FAO activity (Fig. 3j). These results established FAO level as a critical regulator of hepatocyte maturation and indicated that JMJD3 might modulate this process through FAO-dependent mechanisms.

### JMJD3 regulated the level of FAO through HADH

**Fig. 4:**
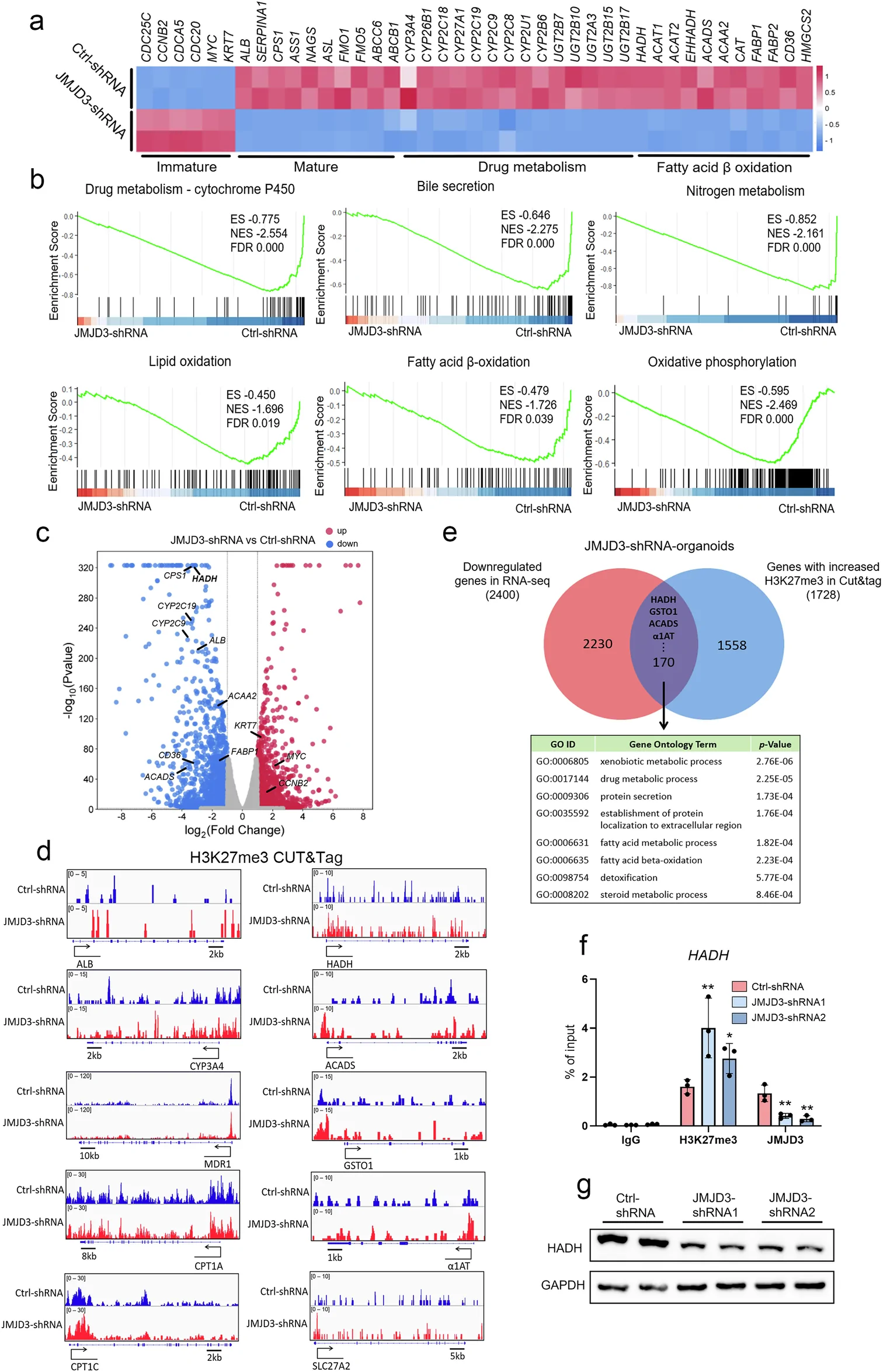
HADH was regulated by JMJD3 through demethylation during hepatocyte maturation. **a** Heatmap for genes related to immature, mature, drug metabolism, and fatty acid oxidation (FAO) in polarized hepatocyte organoids (P-heporgs) treated with ctrl or JMJD3-short hairpin RNAs (shRNAs). **b** Gene Set Enrichment Analysis encoding products related to drug metabolism, bile secretion, nitrogen metabolism, lipid oxidation, FAO, and oxidative phosphorylation, presented as normalized enrichment score (NES). **c** Volcano plot of P-heporgs treated with ctrl or JMJD3-shRNAs. **d** The enrichment of H3K27me3 on hepatocytes and FAO-related genes. **e** Venn diagram for overlap genes between the *n* = 2400 downregulated genes in RNA sequencing (RNA-seq) and *n* = 1728 genes with increased H3K27me3 identified by Cut&Tag. GO term showed the enrichment results of the overlap genes. **f** H3K27me3 and JMJD3 amounts at HADH promoter were assessed by ChIP-qPCR when compared with ctrl-shRNA. **g** The expression of HADH was assessed by western blotting in P-heporgs treated with ctrl or JMJD3-shRNAs. Results were presented as mean ± SD. *P < 0.05, **P < 0.01. ALB, albumin; FDR, false discovery rate.

To delineate the molecular mechanisms underlying JMJD3-mediated hepatocyte maturation, we performed RNA-seq on P-heporgs treated with Ctrl or JMJD3-shRNAs. Transcriptomic analysis revealed that JMJD3 knockdown significantly impaired the expression of genes associated with hepatocyte maturation, drug metabolism, and FAO (Fig. 4a). Gene Set Enrichment Analysis further demonstrated marked downregulation of critical hepatocyte function-related pathways, including cytochrome P450-mediated drug metabolism, bile acid secretion, ammonia detoxification, lipid oxidation, FAO, and oxidative phosphorylation in JMJD3-knockdown organoids (Fig. 4b). These pathways represent essential functional hallmarks of mature hepatocytes, encompassing xenobiotic detoxification, bile acid synthesis and transport, nitrogen metabolism, and lipid metabolic homeostasis. The coordinated suppression of these functional programs indicates that JMJD3 is required not only for the expression of individual maturation markers but also for the establishment of global hepatocyte functional identity. Collectively, these findings demonstrated that JMJD3 broadly regulated multiple core functional programs of mature hepatocytes, including detoxification, bile secretion, and lipid metabolism, thereby establishing its essential role in hepatocyte functional maturation.

Volcano plot analysis of DEGs revealed significant downregulation of hepatocyte functional genes (*CPS1*, *CYP2C9*, *ALB*) and FAO-associated gene *HADH* (false discovery rate < 0.0001) following JMJD3 knockdown (Fig. 4c). *HADH* encodes mitochondrial β-hydroxyacyl-CoA dehydrogenase, a key enzyme in FAO^27^. Given the H3K27me3 demethylase activity of JMJD3, we next examined whether these transcriptional alterations were associated with locus-specific epigenetic changes. H3K27me3 Cut&Tag sequencing was performed in P-heporgs treated with control or JMJD3-shRNAs. Genome-wide profiling revealed pronounced accumulation of H3K27me3 at the promoter regions of *HADH*, *ACADS*, α*1AT*, *GSTO1*, and *SLC27A2* upon JMJD3 knockdown, whereas no significant changes were observed at *ALB*, *CYP3A4*, *MDR1*, *CPT1A*, or *CPT1C* (Fig. 4d), indicating selective locus-specific regulation. To systematically identify potential direct JMJD3 targets, we integrated RNA-seq and H3K27me3 Cut&Tag datasets and defined candidate genes as those exhibiting both decreased mRNA expression and increased promoter H3K27me3 enrichment following JMJD3 knockdown. This analysis identified 170 genes (Fig. 4e). Gene Ontology (GO) enrichment analysis revealed significant over-representation of pathways related to xenobiotic metabolism, drug metabolism, fatty acid metabolic processes, FAO, and detoxification (Fig. 4e and Supplementary Table 4), suggesting the direct epigenetic control of hepatocyte functional programs. ChIP-qPCR further validated the increased H3K27me3 occupancy at *HADH* promoter (Fig. 4f), which correlated with reduced HADH protein expression as demonstrated by WB (Fig. 4g) in JMJD3-knockdown organoids. Collectively, these findings demonstrated that JMJD3 directly regulated a subset of hepatocyte maturation-associated genes through locus-specific H3K27me3 demethylation, with HADH representing a key metabolic target within this regulatory network.

### Acetyl-CoA generated from FAO promoted the maturation of hepatocyte

**Fig. 5:**
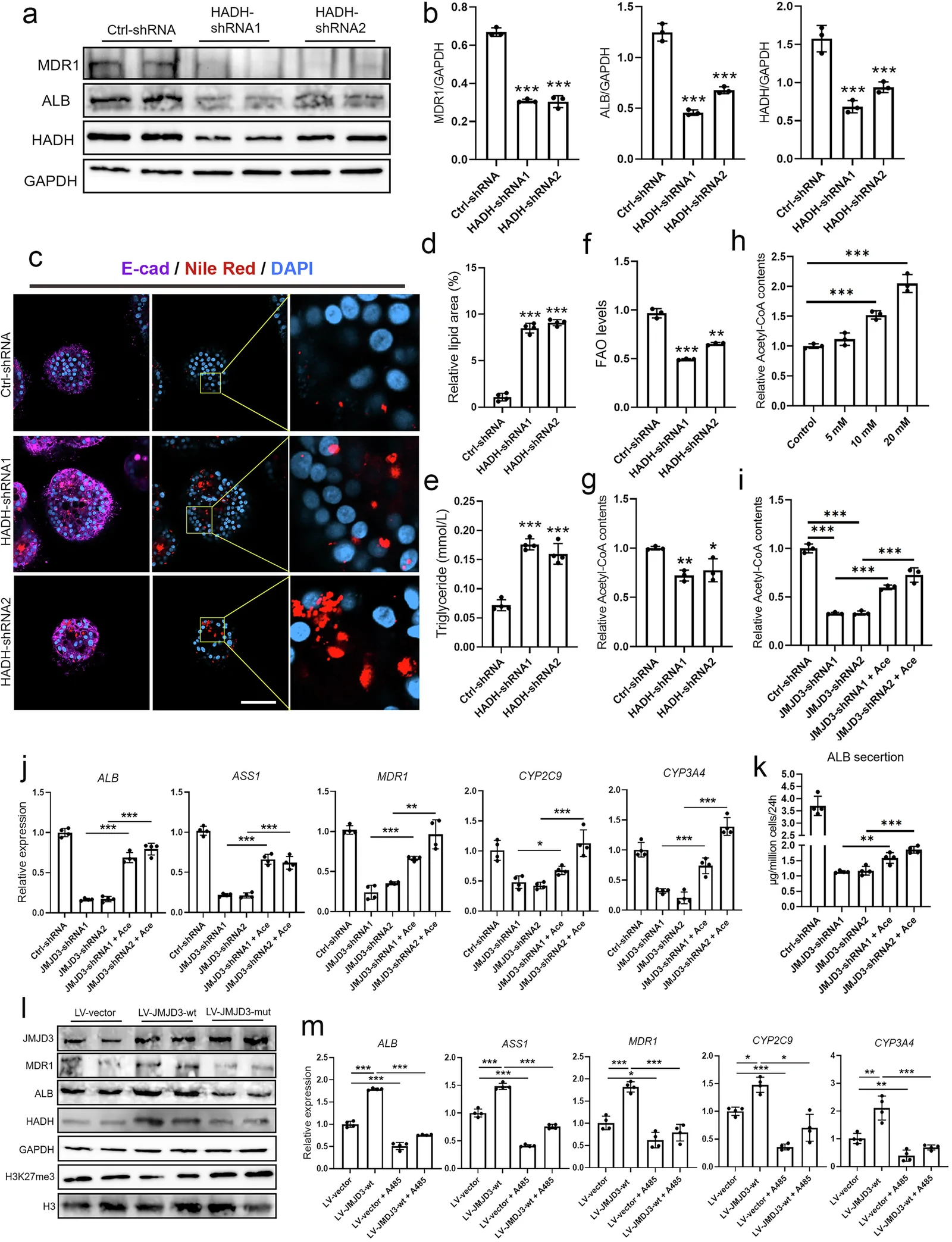
HADH-FAO-mediated histone acetylation regulated the maturation of hepatocytes in P-heporgs. **a** The expressions of MDR1, albumin (ALB), and HADH were assessed by western blotting in polarized hepatocyte organoids (P-heporgs) treated with ctrl or HADH-short hairpin RNAs (shRNAs). **b** The quantification of the expression of MDR1, ALB, and HADH in part **a** when compared with ctrl-shRNA. **c** The representative fluorescent images of Nile Red in P-heporgs treated with ctrl or HADH-shRNAs. Nuclei were stained with 4′,6-diamidino-2-phenylindole (DAPI). Scale bar, 100 μm. The analysis of relative lipid area (part **d**), the levels of fatty acid oxidation (FAO) (part **e**), the content of intracellular triglycerides (part **f**), and the relative content of acetyl-CoA (part **g**) in P-heporgs treated with ctrl or HADH-shRNAs when compared with ctrl-shRNA. **h** The relative content of acetyl-CoA in P-heporgs treated with acetate (ACE). **i** The relative content of acetyl-CoA in P-heporgs treated with ctrl or JMJD3-shRNAs or ACE. **j** The gene expressions related to maturation in P-heporgs treated with ctrl or JMJD3-shRNAs or ACE were analyzed by quantitative reverse transcription (qRT)–PCR. **k** The analysis of secretion of ALB in P-heporgs treated with ctrl or JMJD3-shRNAs or ACE. **l** The expressions of JMJD3, MDR1, ALB, HADH, and H3K27me3 were assessed by western blotting in P-heporgs treated with LV-vector, LV-JMJD3-wt, or LV-JMJD3-mut. **m** The gene expressions related to maturation in P-heporgs treated with LV-vector or LV-JMJD3 or LV-JMJD3-mut or histone acetyltransferase inhibitor (A485) were analyzed by qRT–PCR. Results were presented as mean ± SD. *P < 0.05, **P < 0.01, ***P < 0.001.

To determine the role of HADH in hepatocyte maturation, we constructed Dox-inducible HADH-shRNA lentiviral vector to knockdown HADH during the differentiation of P-heporgs. WB confirmed that HADH knockdown significantly reduced ALB and MDR1 protein levels (Fig. 5a,b). Furthermore, HADH knockdown led to significantly enhanced intracellular lipid deposition and TG content (Fig. 5c–e), concomitant with attenuated FAO activity (Fig. 5f), revealing its essential role in FAO during hepatocyte maturation. Acetyl-CoA, the terminal product of FAO, was markedly diminished in HADH-knockdown organoids (Fig. 5g). Notably, acetate supplementation (a precursor of acetyl-CoA) significantly elevated intracellular acetyl-CoA levels (Fig. 5h) and partially rescued JMJD3 knockdown-induced acetyl-CoA reduction (Fig. 5i). Furthermore, exogenous acetate also restored the suppressed expression of hepatocyte maturation-related genes (*ALB*, *ASS1*, *MDR1*, *CYP2C9*, *CYP3A4*) and the secretion of ALB in both JMJD3-knockdown or HADH-knockdown organoids (Fig. 5j,k and Supplementary Fig. 4a), demonstrating that intracellular acetyl-CoA levels directly affected the maturation of hepatocytes.

To determine whether the demethylase activity of JMJD3 is required for regulating hepatocyte maturation, we generated Dox-inducible lentiviral vectors expressing either wild-type JMJD3 (JMJD3-wt) or its catalytically defective mutant (H1390A, JMJD3-mut) and infected P-heporgs with these constructs. During the differentiation, Dox-induced overexpression of wild-type JMJD3 markedly accelerated the demethylation of H3K27me3 by day 2, whereas the JMJD3-mut failed to reduce H3K27me3 levels (Fig. 5l), confirming disruption of its catalytic activity. Consistent with this epigenetic effect, JMJD3-wt significantly upregulated HADH expression (Fig. 5l), increased FAO activity and intracellular acetyl-CoA content (Supplementary Fig. 4b,c), and enhanced the expression of hepatocyte maturation markers, including MDR1 and ALB (Fig. 5l). By contrast, the JMJD3-mut failed to restore HADH expression, FAO activity, acetyl-CoA production, or maturation-associated protein expression (Fig. 5l and Supplementary Fig. 4b,c). Importantly, the JMJD3-mut was expressed at levels comparable to JMJD3-wt, excluding differences in protein abundance as a confounding factor (Fig. 5l). These results indicated that JMJD3 promoted hepatocyte maturation through its H3K27me3 demethylase activity by activating the HADH-driven FAO pathway, thereby increasing acetyl-CoA availability to support histone acetylation.

Histone acetyltransferases (HATs) are critical regulators of cellular differentiation programs^28,29^. To investigate their potential involvement in JMJD3-mediated hepatocyte maturation, we performed transcriptional profiling of HAT family members during the differentiation of hepatocyte organoids, including *KAT2A*, *KAT2B*, *KAT3B* (EP300), *KAT5*, *KAT6A*, *KAT6B*, *KAT7*, *KAT8*, and *KAT14*. As showed in the heatmap, EP300, *KAT5*, *KAT6A*, *KAT2B*, *KAT6B*, and *KAT8* exhibited stage-specific upregulation during the differentiation (Supplementary Fig. 4d). Notably, EP300 displayed the highest transcriptional expression level and one of the most prominent inductions among analyzed HATs (Supplementary Fig. 4e). Given that EP300 is a well-established H3K27 acetyltransferase critically involved in active enhancer regulation^30^, we further investigated its role during hepatocyte maturation. To determine whether EP300 was responsible for histone acetylation at hepatocyte maturation-related genes under the regulation of JMJD3, we used A485, a selective inhibitor of EP300 acetyltransferase during the differentiation. EP300 inhibition significantly reduced the expression of hepatocyte maturation-associated genes (Fig. 5m) and suppressed the secretion of ALB (Supplementary Fig. 4f). These phenomena were consistently observed in both JMJD3-overexpressing and control P-heporgs, demonstrating that EP300 activity was indispensable for JMJD3-driven maturation. These findings established that JMJD3 orchestrated hepatocyte maturation through an acetyl-CoA-dependent epigenetic mechanism requiring EP300-mediated histone acetylation.

Given the potent lipid metabolic regulatory capacity of JMJD3, we investigated its therapeutic potential in metabolic-associated fatty liver disease (MAFLD) by modeling hepatic steatosis in vitro. P-heporgs were cultured with FFAs (palmitate/oleate, 2:1 ratio) to mimic lipid overload. Strikingly, JMJD3-knockdown organoids under mild lipotoxic conditions (100 μM FFAs) markedly exacerbated intracellular lipid accumulation (Supplementary Fig. 5a). Conversely, JMJD3-overexpression P-heporgs exposed to severe lipotoxic stress (300 μM FFAs) showed significantly reduced lipid deposition (Supplementary Fig. 5b), suggesting that JMJD3 might serve as a potential therapeutic target to alleviate the steatosis in MAFLD. However, further mechanistic and preclinical studies are needed to validate its clinical feasibility.

### JMJD3-mediated H3K27ac regulated the maturation of hepatocytes

**Fig. 6:**
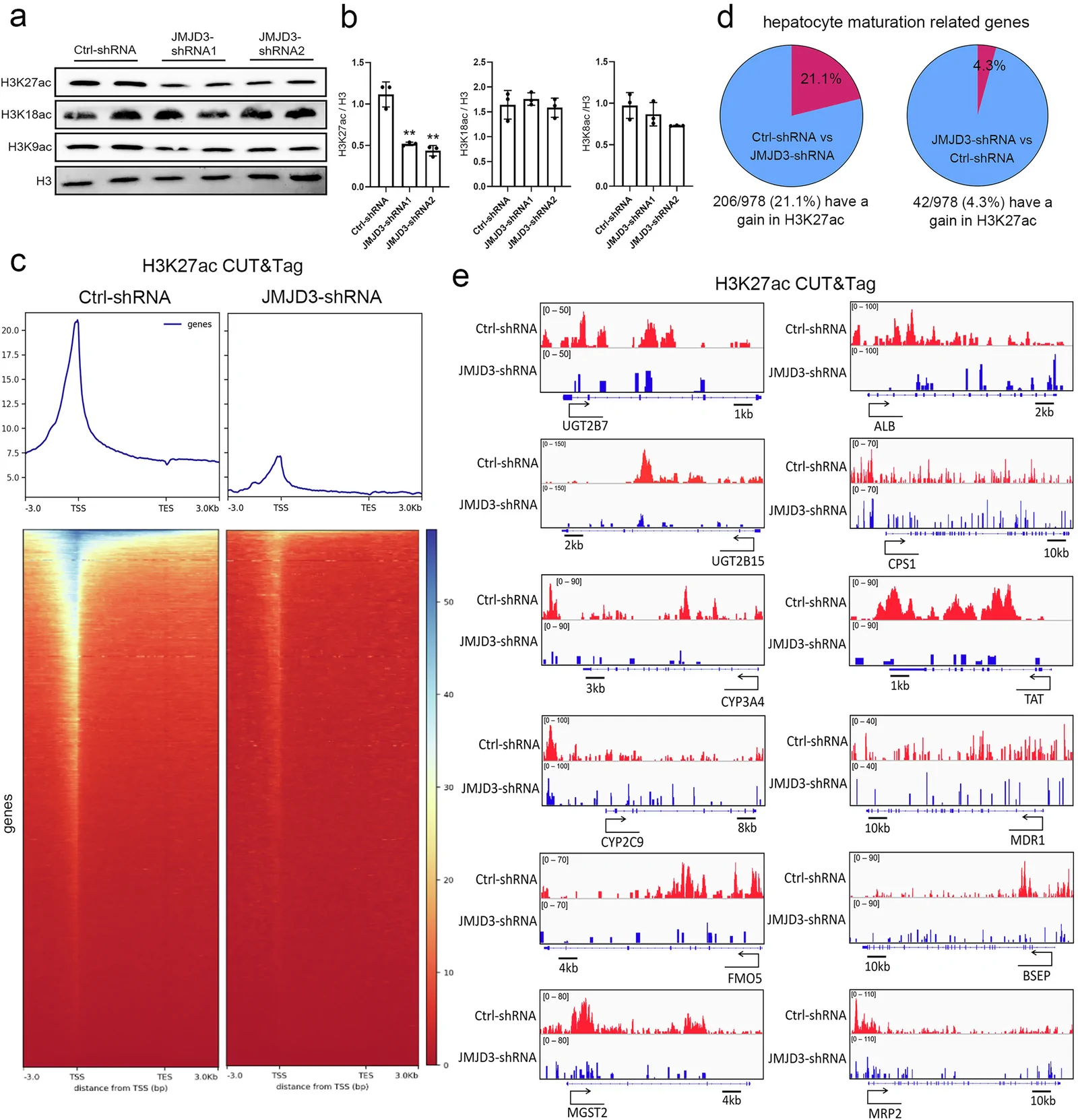
JMJD3-mediated H3K27ac regulated the maturation of hepatocyte in P-heporgs. **a** The expressions of H3K27ac, H3K18ac, and H3K9ac were assessed by western blotting in polarized hepatocyte organoids (P-heporgs) treated with ctrl or JMJD3-short hairpin RNAs (shRNAs). **b** The quantification of the expressions of H3K27ac, H3K18ac, and H3K9ac, H3 was used as a loading control when compared with ctrl-shRNA. **c** The average plots and heatmaps of H3K27ac CUT&Tag signals at transcriptional start sites (TSSs) between P-heporgs treated with ctrl-shRNA or JMJD3-shRNA. **d** Proportions of hepatocyte maturation-related genes with enhanced H3K27ac (red portion) in ctrl-shRNA treatment versus JMJD3-shRNA treatment. **e** The enrichment of H3K27ac on hepatocyte maturation-related genes. Results were presented as mean ± SD. **P < 0.01.

To elucidate the association between JMJD3 and histone acetylation during hepatocyte maturation, we investigated acetyltransferase p300-mediated histone acetylation targets following JMJD3 knockdown. WB analysis revealed a specific decrease in H3K27ac levels in JMJD3-knockdown organoids, whereas H3K9ac and H3K18ac remained unchanged (Fig. 6a,b), indicating that JMJD3 selectively regulated H3K27ac modification. To gain deeper insights into the changes in histone acetylation, Cut&Tag was performed to compare genome-wide changes in H3K27ac between Ctrl-shRNA P-heporgs and JMJD3-shRNA P-heporgs. The Cut&Tag analyses showed H3K27ac intensities around transcriptional start sites (TSSs) that were prominently declined in JMJD3-knockdown P-heporgs (Fig. 6c). The changes in H3K27ac at promoter or enhancer regions of hepatocyte maturation-associated gene sets showed a greater enrichment of H3K27ac in Ctrl-shRNA P-heporgs (206/978, 21.1%) compared with JMJD3-shRNA P-heporgs (42/978, 4.3%) (Fig. 6d), including key hepatocyte maturation-related genes (*ALB*, *CPS1*, *TAT*), polarization-related genes (*MDR1*, *BSEP*, *MRP2*), and drug metabolism-related genes (*UGT2B7*, *UGT2B15*, *CYP3A4*, *CYP2C9*, *FMO*, *MGST2*) (Fig. 6e).

To determine whether the reduction in global H3K27ac upon JMJD3 knockdown is mediated through regulation of the HAT EP300, we examined EP300 expression at both the transcriptional and protein levels. RNA-seq analysis revealed that *EP300* mRNA levels were not decreased but instead moderately increased in JMJD3-knockdown P-heporgs (Supplementary Fig. 6a). Consistently, WB analysis confirmed that EP300 protein levels were also elevated following JMJD3 depletion (Supplementary Fig. 6b). These results indicated that the reduction in global H3K27ac was unlikely to result from decreased EP300 expression and therefore did not support a model in which JMJD3 directly promoted H3K27ac through transcriptional regulation of EP300. Collectively, these findings demonstrated that the loss of JMJD3 resulted in the decrease in H3K27ac levels at hepatocyte maturation-associated genes during the differentiation of hepatocyte organoids.

### JMJD3 regulated the maturation of hepatocytes in mice fetal liver and 2D differentiated hepatocytes

**Fig. 7:**
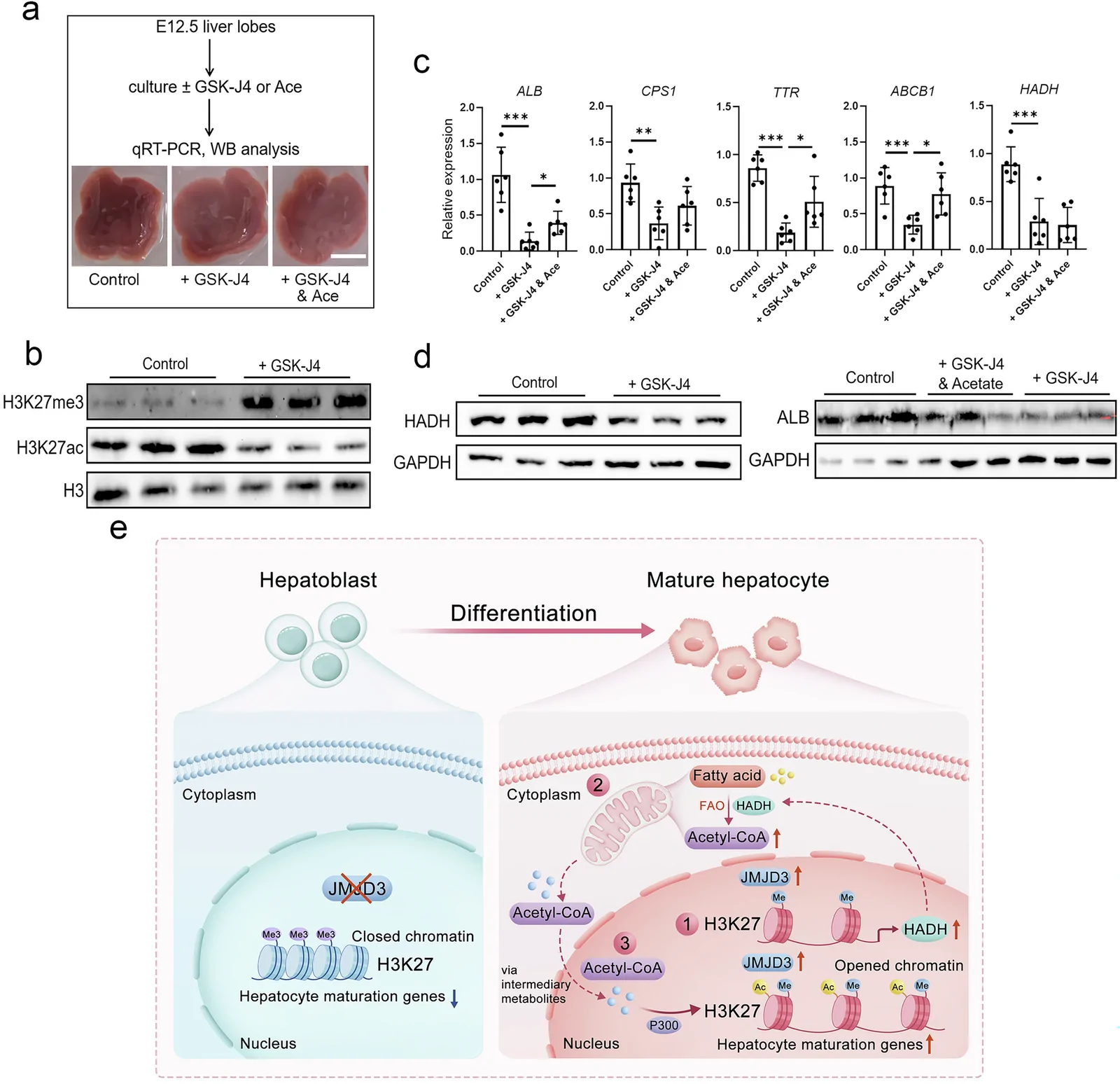
JMJD3 regulated the maturation of hepatocyte in mice. **a** Schematic representation of E12.5 mouse liver lobe culture and images of cultured liver lobes. Scale bar, 100 μm. **b** The expressions of H3K27me3 and H3K27ac were assessed by western blotting (WB) in mouse liver lobe treated with GSK-J4. *n* = 3 mouse liver lobes. **c** The gene expressions related to hepatocyte maturation in mouse liver lobes treated with GSK-J4 or acetate were analyzed by quantitative reverse transcription–PCR (qRT–PCR). *n* = 6 mouse liver lobes. **d** The expressions of HADH and albumin (ALB) were assessed by WB in mouse liver lobes treated with GSK-J4 or acetate. *n* = 3 mouse liver lobes. **e** Schematic diagram showing the process of JMJD3 regulating the maturation of hepatocyte. Briefly, JMJD3 activated HADH by the demethylation (stage 1), then HADA promoted fatty acid oxidation (FAO) to generate acetyl-CoA (stage 2), eventually acetyl-CoA-dependent P300-mediated H3K27 acetylation promoted hepatocyte maturation (stage 3). Results were presented as mean ± SD. *P < 0.05, **P < 0.01, ***P < 0.001.

To further investigate the functions of JMJD3 in regulating hepatocyte maturation, we isolated E12.5 mouse fetal livers and cultured them with the inhibitor of JMJD3, 10 μM GSK-J4 (Fig. 7a). Forty-eight hours after the culture, GSK-J4 treatment induced genome-wide H3K27me3 accumulation concomitant with H3K27ac depletion in fetal livers (Fig. 7b), while maintaining morphological and dimensional characteristics comparable to control group (Fig. 7a). qRT–PCR analyses revealed that fetal livers treated with GSK-J4 showed decreased expression of *HADH* and maturation-related genes *ALB*, *CPS1*, *TTR,* and *ABCB1*, whereas the addition of acetate partially rescued the expression of these genes (Fig. 7c). WB analyses further confirmed the decreased expression of HADH and ALB on protein levels (Fig. 7d). These results demonstrated that JMJD3–HADH–FAO–H3K27ac axis was indeed effective in regulating the maturation of hepatocytes.

To further determine whether the JMJD3–HADH–FAO axis also functions during hepatocyte differentiation under conventional 2D culture conditions, we performed validation experiments using the H9 hESC-derived hepatocyte differentiation system. Morphological changes during the differentiation process from hESCs to HLCs were confirmed (Supplementary Fig. 7a). qRT–PCR analysis revealed that the expression of hepatocyte maturation-associated genes, including *ALB*, *CYP3A4*, *CYP2C9*, *MDR1, HADH* and *JMJD3*, and *UTX*, was significantly increased during the maturation from hepatoblasts to HLCs (Supplementary Fig. 7b). GSK-J4 treatment significantly reduced FAO activity (Supplementary Fig. 7c), accompanied by decreased ALB secretion (Supplementary Fig. 7d) and downregulation of hepatocyte maturation-related genes (Supplementary Fig. 7e). Importantly, acetate supplementation partially rescued ALB secretion and the expression of hepatocyte functional genes under GSK-J4 treatment. These findings indicated that the JMJD3–HADH–FAO axis may represent a general regulatory pathway governing hepatocyte maturation across different differentiation systems.

Taken together, our results demonstrated that JMJD3 orchestrated hepatocyte maturation through a coordinated epigenetic-metabolic cascade. During the differentiation of hESC-derived hepatocyte organoids, JMJD3 mediated H3K27me3 demethylation at the FAO-critical enzyme HADH and a subset of maturation-associated genes, thereby epigenetically activating their expression. HADH-encoded β-hydroxyacyl-CoA dehydrogenase catalyzed hydroxyacyl-CoA dehydrogenation to promote FAO flux, generating acetyl-CoA which served as the substrate for EP300-mediated H3K27 acetylation at loci of hepatocyte maturation genes, thereby enhancing their transcriptional activation and driving hepatocyte maturation (Fig. 7e).

## Discussion

Although epigenetic modifications and metabolic remodeling are recognized as critical determinants of cellular differentiation, the regulatory interplay between epigenetic regulators and FAO-dependent lipid metabolism during hepatocyte maturation remains poorly defined. In this study, we systematically mapped dynamic changes in epigenetic regulator expression and lipid metabolic profiles during the maturation of hESC-derived hepatocyte organoids and explored their roles through pharmacological and genetic interventions. Specifically, we found that FAO levels had a regulatory effect on hepatocyte maturation, and it was regulated by epigenetic regulator JMJD3 through histone demethylation on HADH, the key enzyme of FAO. Mechanistically, the upregulated FAO activity promoted hepatocyte maturation by accumulating acetyl-CoA levels and increased the acetylation of H3K27 on hepatocyte maturation-related genes. Our study illustrated an unrevealed regulation and synergistic effect between epigenetic modification and FAO in human hepatocyte maturation.

Although hepatocyte differentiation has been extensively characterized in the context of transcription factors, signaling pathways, non-coding RNAs, and epigenetic mechanisms, the synergistic interplay between metabolic and epigenetic regulations during human hepatocyte maturation remains poorly understood. Cellular differentiation is inherently coupled to metabolic remodeling, with distinct metabolic signatures emerging across lineages. For instance, PSCs predominantly utilize glycolysis, whereas endoderm specification coincides with a shift toward oxidative phosphorylation, whereas ectodermal lineages retain elevated glycolytic activity, and the metabolic remodeling is critical for the differentiation of early ectoderm and endoderm^31^. Pioneer works have illustrated that the high level of FAS is maintained during the initiation of liver regeneration^32,33^ and before liver maturation^34^. Consistent with these precedents, our HB-orgs exhibited the robust FAS activity, whereas P-heporgs displayed a marked upregulation of FAO (Fig. 3a–d). This metabolic reprogramming aligns with the reliance of functionally mature hepatocytes on FAO to support their biosynthetic and detoxification roles^35^. Despite these evidence, the mechanistic coupling between FAO dynamics and hepatocyte maturation remains unexplored. Our study confirmed the FAO activity as a critical determinant of the regulation on hepatocyte maturation (Fig. 3g–j) through FAO-generated acetyl-CoA-mediated H3K27 acetylation in differentiated organoid model (Fig. 7e). Importantly, mitochondrial acetyl-CoA generated from FAO contributes to cytoplasmic and nuclear acetyl-CoA pools through intermediary metabolites such as citrate or acetate^36^. Although our findings support a functional link between FAO-derived acetyl-CoA metabolism and histone acetylation during hepatocyte maturation, the precise routes by which acetyl-CoA is transferred to the nucleus remain to be fully elucidated. Future studies combining compartment-resolved metabolomics and isotope tracing approaches would help further define the metabolic pathways connecting FAO to histone acetylation during hepatocyte maturation^37^.

In addition, acetate supplementation was found to partially rescue the maturation defects induced by JMJD3 inhibition in this study. Although acetate replenishes acetyl-CoA and restores histone acetylation downstream of FAO, it does not reverse the accumulation of repressive H3K27me3 marks caused by loss of JMJD3 demethylase activity. Moreover, FAO contributes to broader metabolic processes, including mitochondrial function and redox homeostasis, which are essential for hepatocyte maturation but cannot be restored by acetate alone. Therefore, these findings suggest that JMJD3 regulates hepatocyte maturation through coordinated epigenetic mechanisms and FAO-dependent metabolic control, further supporting its dual role in integrating chromatin regulation and cellular metabolism.

Consistent with our findings, previous studies have demonstrated that JMJD3 expression is markedly reduced in human steatotic liver samples and that JMJD3 deficiency exacerbates hepatic lipid accumulation by impairing hepatic autophagy and lipid degradation^38,39^. These observations, together with our results, support a conserved role for JMJD3 as a key epigenetic regulator of FAO and hepatic lipid homeostasis. In line with this notion, our study demonstrated that JMJD3 knockdown suppressed multiple FAO-related genes, including *HADH*, *ACADS*, *ACAA2*, and *EHHADH*, leading to impaired FAO activity and increased lipid accumulation. Conversely, JMJD3 overexpression alleviated lipid deposition under lipotoxic stress conditions, further supporting a protective role for JMJD3 in maintaining hepatic lipid homeostasis during metabolic stress. Collectively, these findings highlight the translational relevance of the JMJD3–FAO axis and support further investigation of this pathway as a potential therapeutic target for MAFLD. Although CPT1A is widely regarded as the rate-limiting enzyme of mitochondrial fatty acid oxidation, HADH functions as an essential enzyme within the β-oxidation cycle by catalyzing the third step of FAO. Consistent with previous studies showing that HADH deficiency impairs fatty acid oxidation and disrupts metabolic homeostasis^40^, our findings demonstrated that HADH knockdown markedly reduced FAO flux, supporting the conclusion that JMJD3 promoted hepatocyte maturation by transcriptionally activating HADH and sustaining FAO-dependent acetyl-CoA production.

The spatiotemporally precise dynamics of H3K27me3 modifications orchestrate the rigorous progression of organogenesis during development^8,41,42^. Previous studies in murine models revealed that the loss of enhancer of zeste homolog (EZH1/2) induced the premature hepatocyte differentiation from postnatal day 14 with decreased H3K27me3 on hepatocyte maturation genes^43^, which is similar to our findings that the overexpression of JMJD3 accelerated the maturation of hepatocyte organoids (Fig. 5l). Our results also indicated that JMJD3 affected FAO through direct demethylation on H3K27me3 of HADH and further regulated hepatocyte maturation (Fig. 7e). Collectively, these studies established JMJD3-mediated H3K27me3 modulation as a temporal regulator of hepatocyte maturation and lipid metabolic homeostasis.

Although both JMJD3 and UTX catalyze H3K27me3 demethylation, their functional roles during the development are highly context-dependent. Previous studies have shown that UTX is essential for differentiation of specific retinal neurons^44^, whereas JMJD3 has a dominant role in CD8⁺ T cell differentiation^45^, and both enzymes contribute to neural progenitor lineage specification in a non-redundant manner^25^. Consistent with this context-specific functional specialization, our results demonstrated that JMJD3, but not UTX, was required for hepatocyte maturation, identifying JMJD3 as the principal H3K27me3 demethylase regulating hepatocyte lineage progression. Previous work by Pediconi et al.^46^ similarly demonstrated in a HepRG differentiation model that JMJD3 was required for hepatocyte maturation. Importantly, despite differences in experimental systems, both studies consistently demonstrate that JMJD3 activity is essential for hepatocyte maturation. Furthermore, our study extends these findings by uncovering a mechanistic link between JMJD3-mediated H3K27me3 demethylation and FAO-dependent metabolic remodeling, establishing the JMJD3–FAO–acetyl-CoA axis as a key regulatory pathway driving hepatocyte maturation.

Although PSC-derived hepatocyte organoids faithfully model key aspects of human hepatocyte development in vitro, current platforms remain limited in recapitulating the liver’s intricate multicellular architecture and physiological microenvironment. As the primary metabolic organ, the liver possesses a highly specialized polarized architecture supported by complex interactions among parenchymal and non-parenchymal cell populations^47^. These cellular components originate from distinct embryonic lineages^48^, including endoderm-derived hepatocytes and mesoderm-derived hepatic stellate and endothelial cells, thereby creating substantial challenges for current hepatocyte organoid systems to faithfully recapitulate native liver tissue organization and multicellular complexity. By contrast, some other organoid systems, such as intestinal organoids, primarily arise from a common epithelial lineage and therefore exhibit a greater capacity for lineage-driven self-organization^49^. Although mature hepatocyte-derived organoids can recapitulate multipolarized features, these models still lack critical non-parenchymal cell populations, including hepatic stellate cells, Kupffer cells, and liver sinusoidal endothelial cells^50,51^. These non-parenchymal cells are known to have essential roles in hepatocyte differentiation, metabolic regulation, tissue remodeling, and disease progression through extensive cell–cell communication and paracrine signaling^52^. Similarly, although ex vivo fetal liver culture model preserves aspects of native tissue architecture and cellular heterogeneity, it cannot fully recapitulate the complex physiological and metabolic microenvironment of fetal or adult liver in vivo. Recently, Hofer et al.^53^ developed a human gastric organoid-on-chip system that faithfully reproduced the architectural features and cellular diversity of native human antral tissue, providing important conceptual and technical insights for future liver modeling strategies. By integrating biomimetic engineering approaches, hydrogel-supported tissue engineering platforms, multicellular co-culture systems, and organoid technologies, more physiologically relevant liver models with improved cellular diversity and tissue organization may be established. In addition, future studies using hepatocyte-specific JMJD3 conditional knockout mice will further validate the physiological relevance of JMJD3–FAO axis in vivo^38,39^.

In conclusion, we dissected a crucial role for JMJD3-mediated regulation of hepatocyte maturation in hESC-derived organoids. Our results not only revealed the role of JMJD3-mediated epigenetic regulation in hepatocyte differentiation but also provided the evidence of the important role of FAO-mediated lipid metabolism in hepatocyte maturation.

## Supplementary information

Supplementary Information

## Data Availability

The data that support the findings of this study are available from the corresponding author upon request. The RNA-seq and Cut&Tag datasets have been deposited in GEO with accession numbers GSE295093 and GSE297175.
