# Supplementary material for: JMJD3-mediated HADH demethylation orchestrates hepatocyte maturation through fatty acid β-oxidation-dependent histone acetylation
Source: Exp Mol Med. 2026 Sep 1;58(9):2888–902. doi: 10.1038/s12276-026-01816-x (PMC13633256; doi:10.1038/s12276-026-01816-x)
Supplement: Supplementary file 1 [file 12276_2026_1816_MOESM1_ESM.pdf]

Supplementary Information

JMJD3 mediated-HADH demethylation orchestrates hepatocyte maturation through fatty acid β-oxidation-dependent histone acetylation

Haibin Wu[1,2,3], Sen Chen[1,2,3], Huanhuan Shan[1,2,3], Wenjiao Yan[1,2,3], Changlu Qin[1,2,3], Shoupei Liu[1,2,3], Xiangting Cao[1,2,3], Jinzhi Du[4], Yongjian Zhou[1], Yuyou Duan[1,2,3,5,6]*

Affiliations:

[1]Department of Gastroenterology and Hepatology, Guangzhou Digestive Disease Center, the Second Affiliated Hospital, School of Medicine, South China University of Technology; Guangzhou, 510006, China.

[2]Laboratory of Stem Cells and Translational Medicine, Center for Medical Research on Innovation and Translation, Institute of Clinical Medicine, the Second Affiliated Hospital, School of Medicine, South China University of Technology; Guangzhou, 510006, China.

[3]Laboratory of Stem Cells and Translational Medicine, Institute for Life Science, School of Medicine, South China University of Technology; Guangzhou, 510006, China.

[4]School of Medicine, South China University of Technology; Guangzhou, 510006, China.

[5]National Engineering Research Center for Tissue Restoration and Reconstruction, South China University of Technology; Guangzhou, 510006, China.

[6]The Innovation Centre of Ministry of Education for Development and Diseases, the Second Affiliated Hospital, School of Medicine, South China University of Technology; Guangzhou 510006, China.

*Corresponding author: Email: yuyouduan@scut.edu.cn

Supplementary figures:

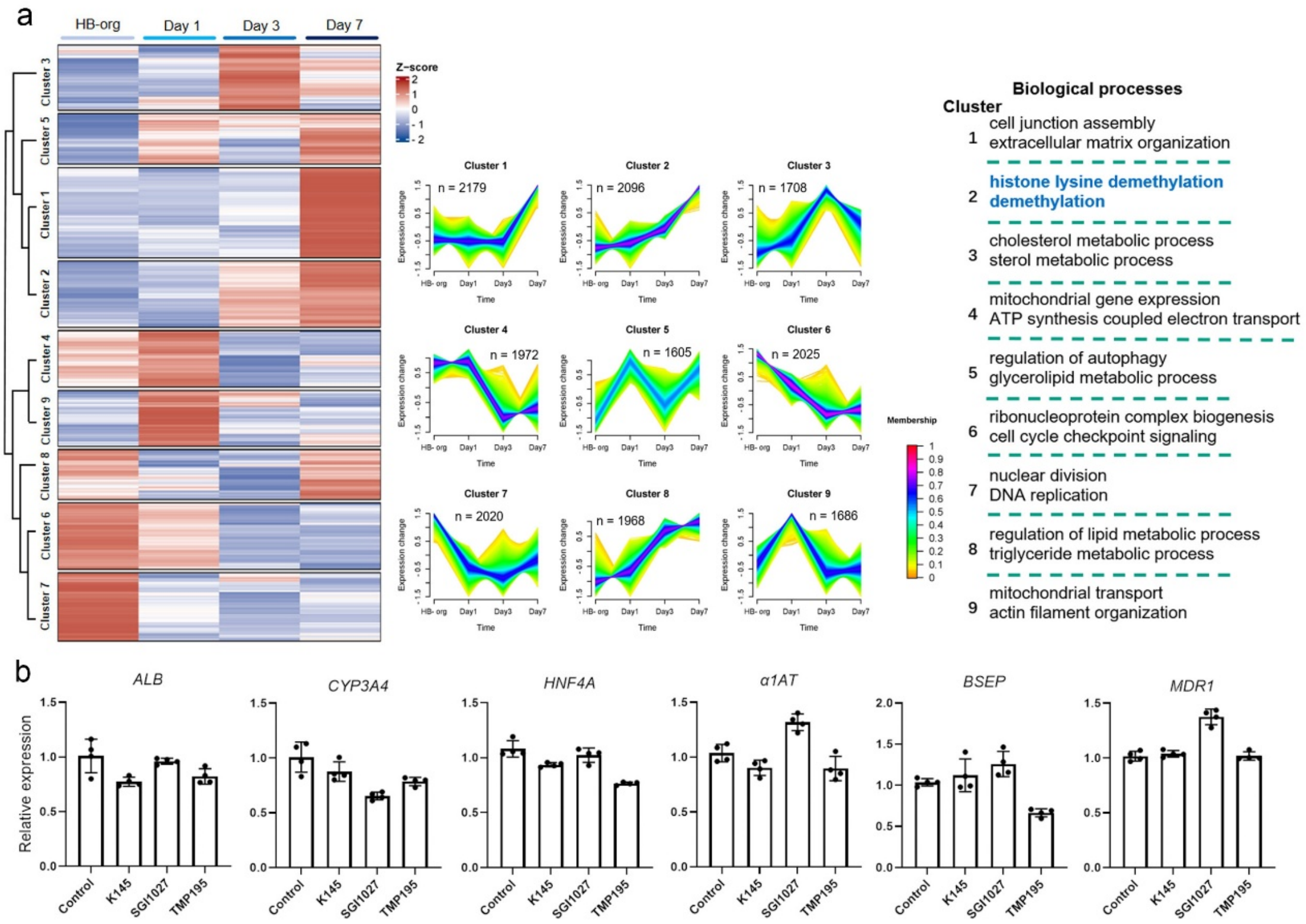

Supplementary Fig. 1. Exploration of key regulatory factors of hepatocyte maturation

(a) The complete results of hierarchical clustering of all DEGs in HB-orgs and P-heporgs at 3 time points. The heatmap represented the average transcripts-per-million (TPM) values of biological replicates scaled per gene (z-score). The clusters were numbered as 1–9; n, the number of genes per cluster. Gene ontology (GO) analyses were performed. (b) The expressions of genes related to hepatocyte maturation in P-heporgs treated with different inhibitors for epigenetic regulatory factors were analyzed by qRT-PCR (K145, SPHK1/2 inhibitor; SGI1027, DNMT3A inhibitor; TMP195, HDAC7 inhibitor). Results were presented as mean ± SD.

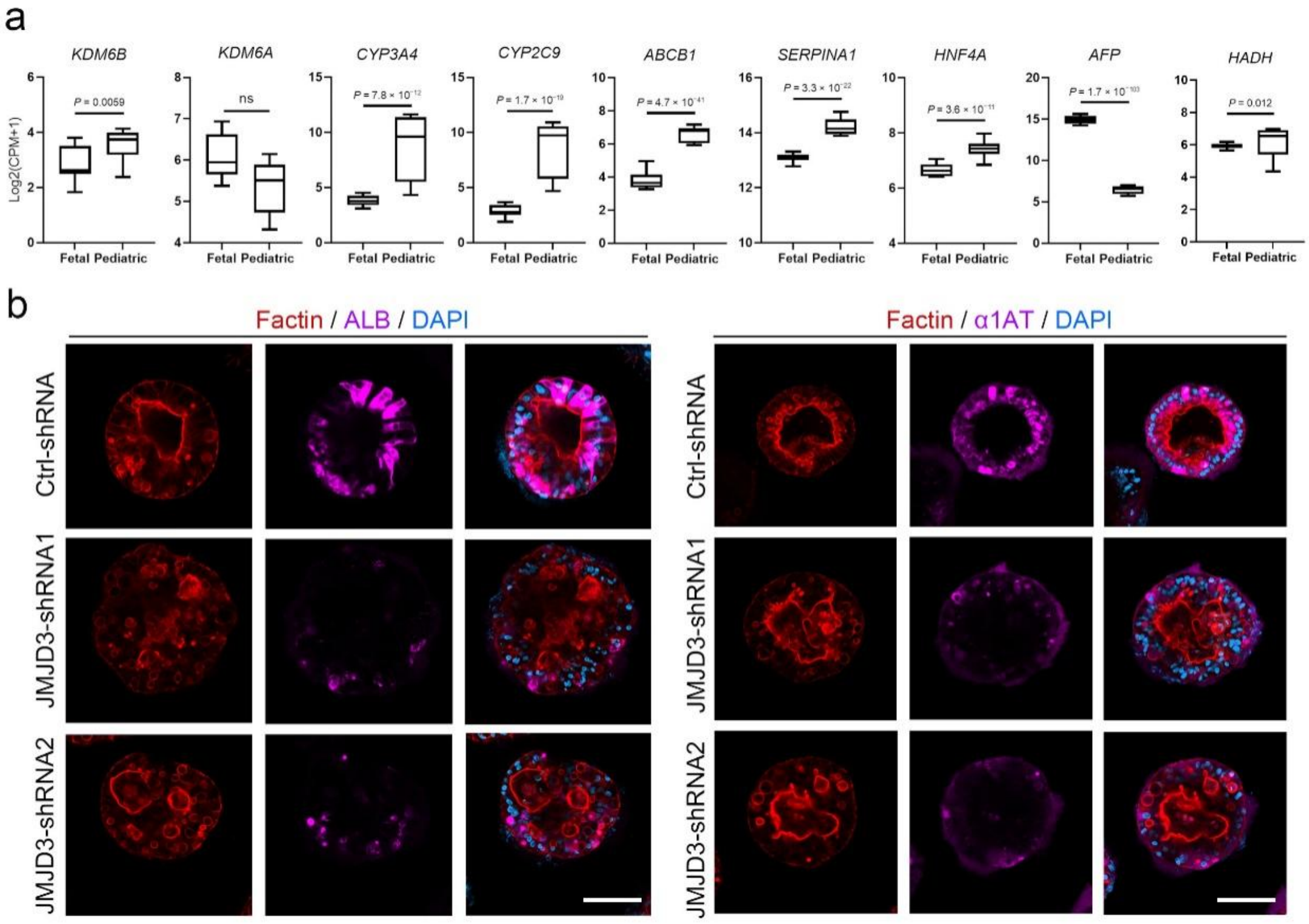

Supplementary Fig. 2. JMJD3 affected the maturation of hepatocytes

(a) Differential expression of hepatocyte maturation-related genes between fetal and pediatric liver samples (n=10). Statistical significance was determined using edgeR differential expression analysis. (b) Immunostaining for Factin and ALB or α1AT in P-heporgs treated with ctrl or JMJD3-shRNAs. Nuclei were stained with DAPI. Scale bar = 100 μm.

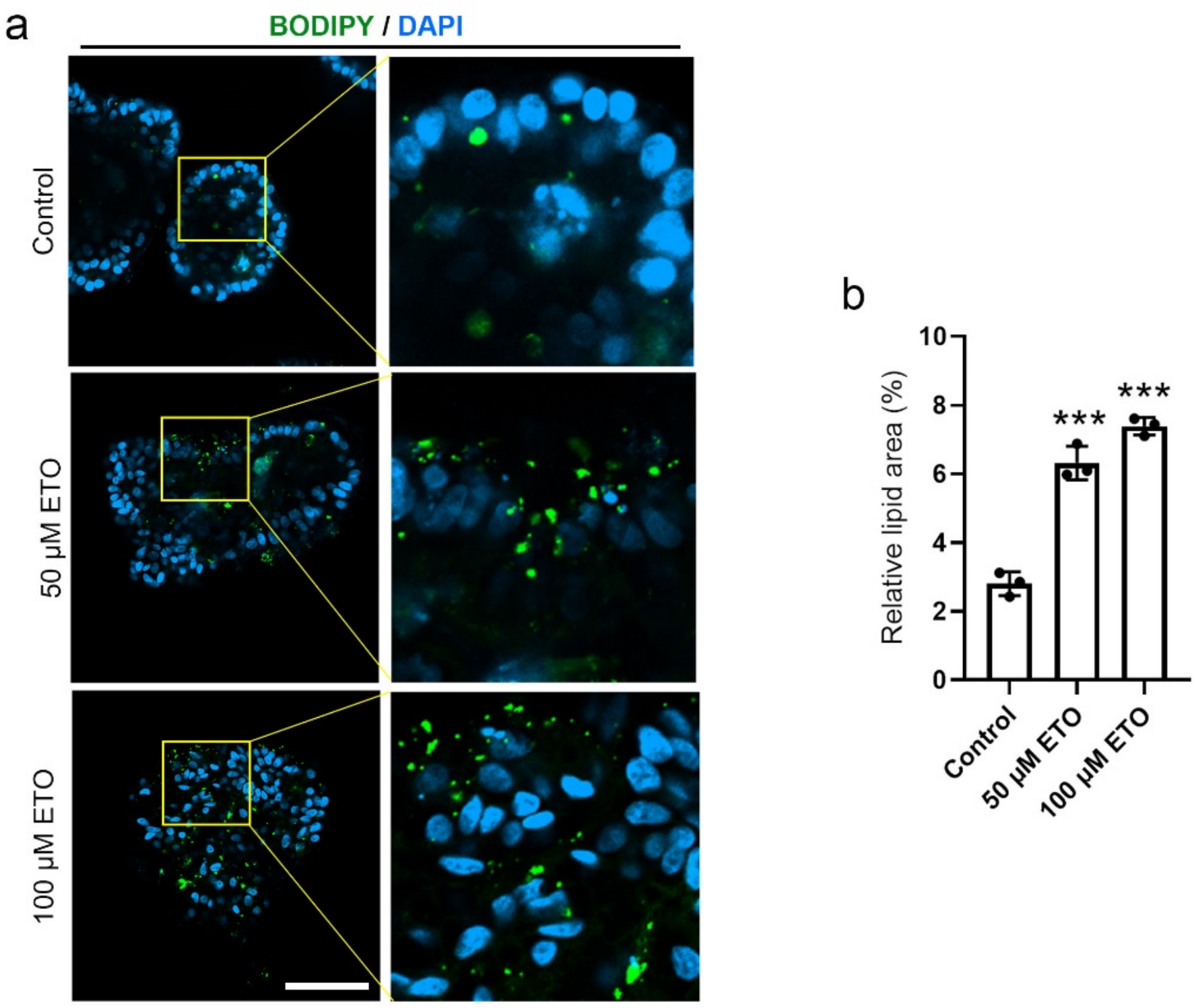

Supplementary Fig. 3. FAO levels affected lipid accumulation in hepatocytes of P-heporgs

(a) The representative fluorescent images of BODIPY in P-heporgs treated with or without ETO. Nuclei were stained with DAPI. Scale bar = 100 μm. (b) The analysis of relative lipid area in P-heporgs treated with or without ETO when compared with control. Results were presented as mean ± SD. ****p* <0.001.

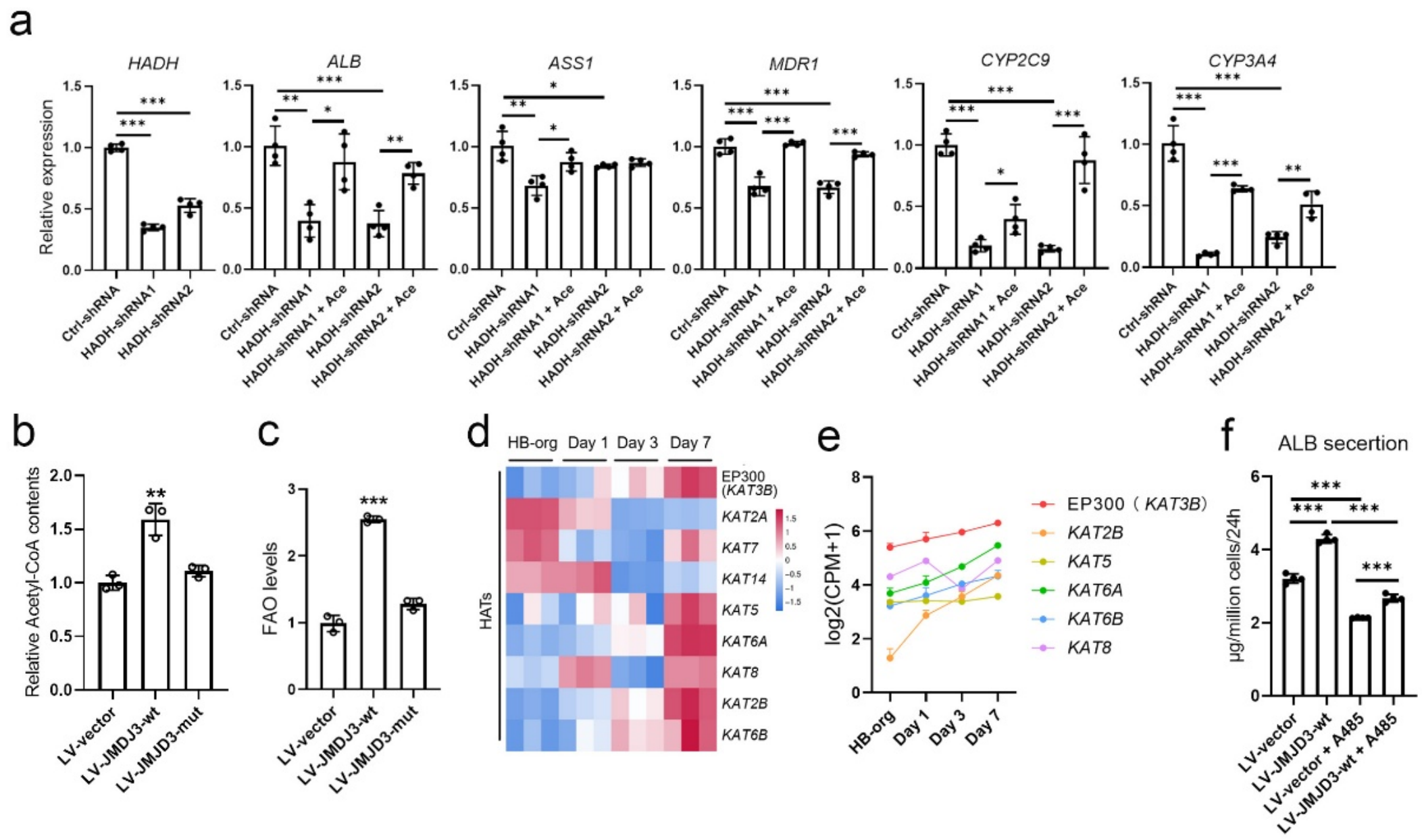

Supplementary Fig. 4. FAO levels affected lipid accumulation in hepatocytes of P-heporgs

(a) The expressions of genes related to hepatocyte maturation in P-heporgs treated with ctrl or HADH-shRNAs or acetate were analyzed by qRT-PCR. (b, c) The content of acetyl-CoA (b) and FAO level (c) was assessed in indicated groups when compared with LV-vector. (d) Heatmap for genes related to KATs. (e) The log_2 (TPM) expression of KATs. (f) The analysis of the secretion of ALB by P-heporgs transduced with LV-vector or LV-JMJD3-overexpression. Results were presented as mean ± SD. *p <0.05, **p <0.01, ***p <0.001.

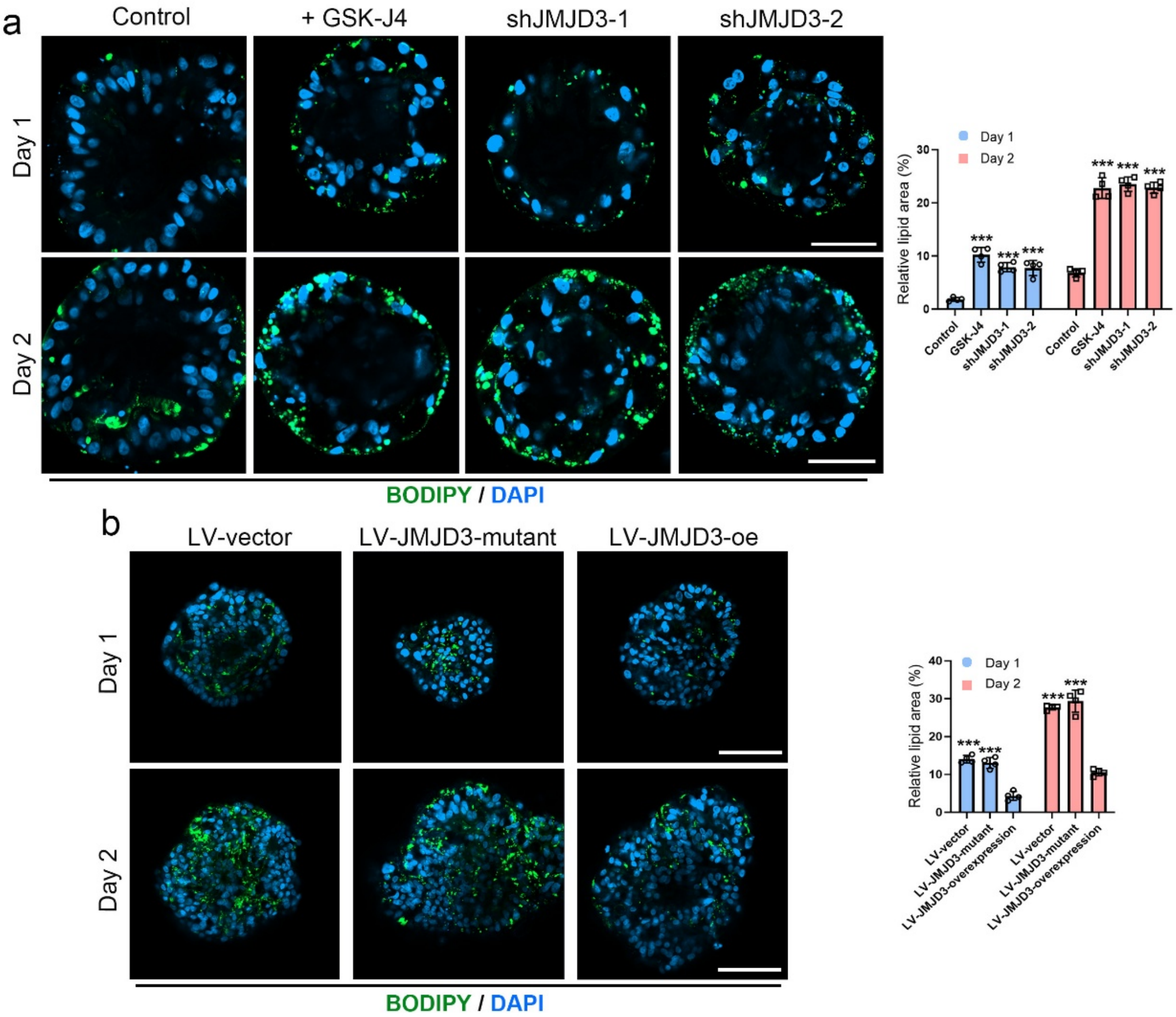

Supplementary Fig. 5. FAO levels affected lipid accumulation in hepatocytes of P-heporgs

(a) The representative fluorescent images of BODIPY staining and relative fluorescent area in P-heporgs treated with GSK-J4 or JMJD3-shRNA when compared with control. Nuclei were stained with DAPI. Scale bar = 50 μm. (b) The representative fluorescent images of BODIPY staining and relative fluorescent area in P-heporgs transduced with LV-vector, LV-JMJD3-mutant or LV-JMJD3-overexpression. Nuclei were stained with DAPI. Scale bar = 100 μm. Results were presented as mean ± SD. ***p <0.001.

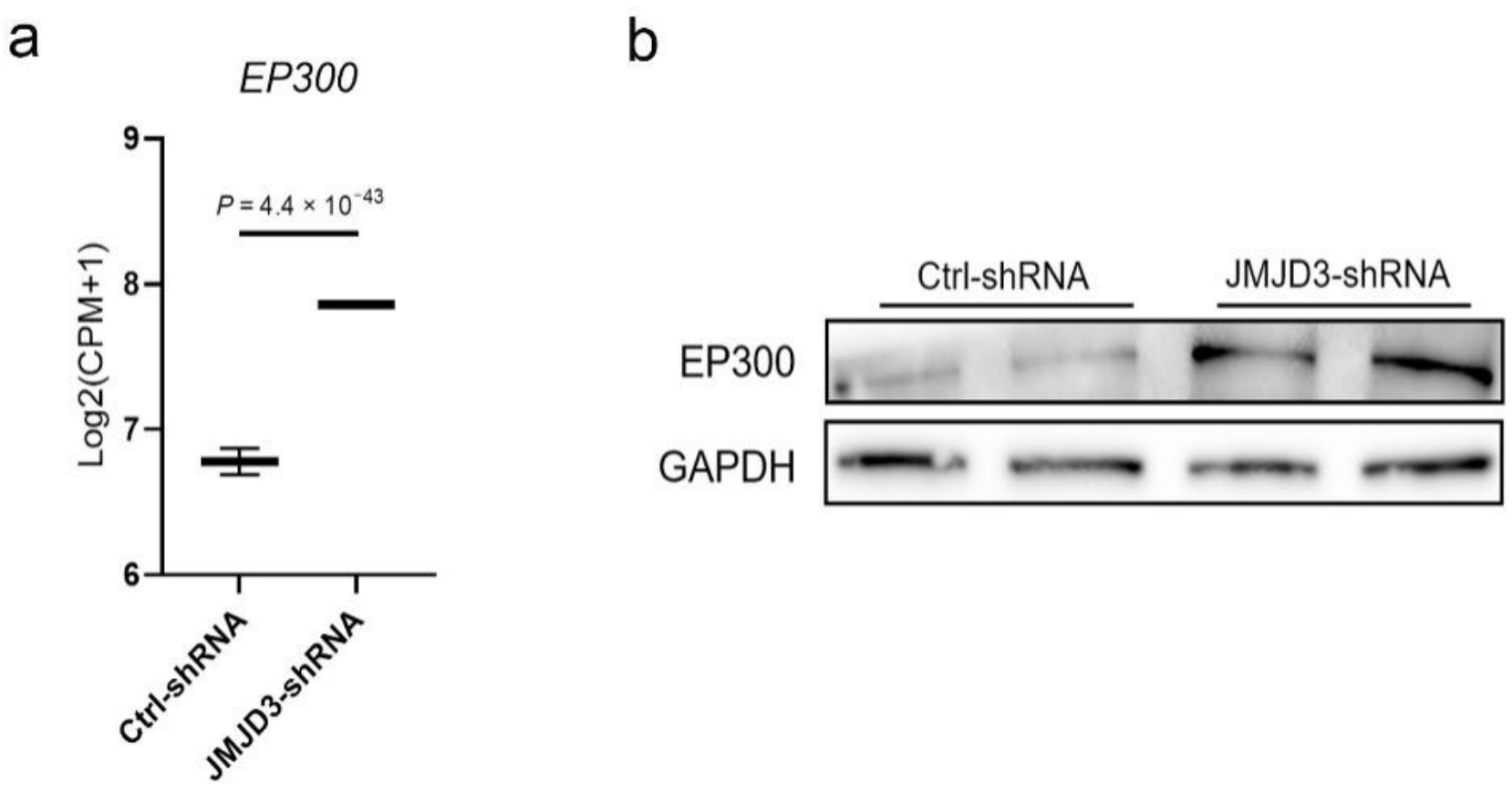

Supplementary Fig. 6. EP300 expression was not decreased in JMJD3-knockdown hepatocyte organoids

(a) Differential expression of *EP300* between Ctrl- and JMJD3-shRNA treated organoids. Statistical significance was determined using edgeR differential expression analysis. (b) The expression of EP300 was assessed by WB in P-heporgs treated with Ctrl- and JMJD3-shRNA.

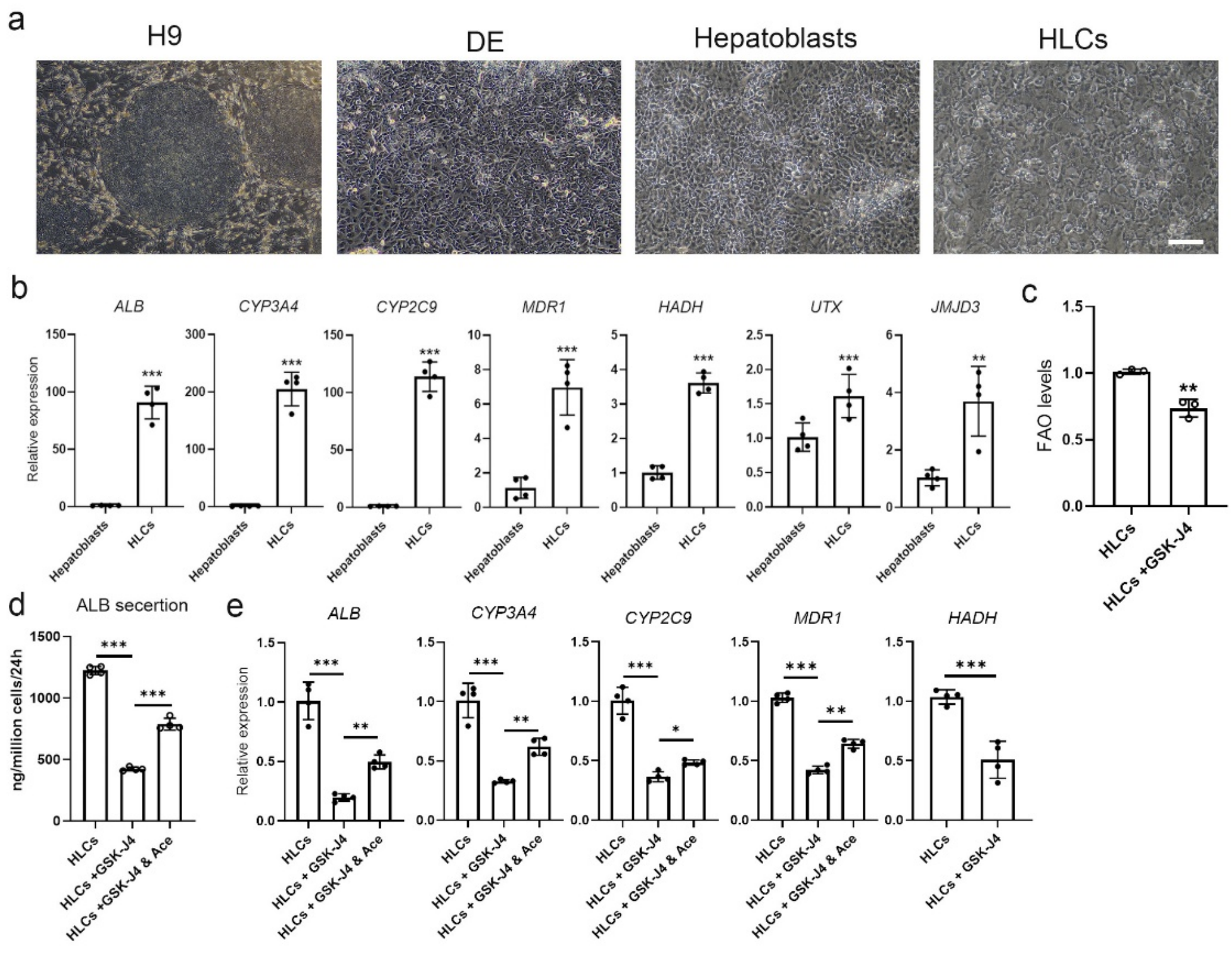

Supplementary Fig. 7. JMJD3 regulated the maturation of 2D differentiated hepatocytes

(a) Representative morphological images during the differentiation of H9 cells into hepatocyte-like cells (HLCs) through definitive endoderm (DE) and hepatoblast stages under conventional 2D culture conditions. Scale bar, 100 μm. (b) The expressions of genes related to hepatocyte maturation (*ALB*, *CYP3A4*, *CYP2C9*, *MDR1*, and *HADH*) and H3K27 demethylases (*UTX* and *JMJD3*) in differentiated hepatoblasts and HLCs were analyzed by qRT-PCR. (c) The assessment of FAO levels in HLCs treated with or without GSK-J4. (d) The quantification of ALB secretion in HLCs treated with GSK-J4 or GSK-J4 plus acetate supplementation. (e) The expressions of genes related to hepatocyte maturation in HLCs treated with GSK-J4 or GSK-J4 plus acetate supplementation were analyzed by qRT-PCR. Results were presented as mean ± SD. *p

<0.05, ** $p<0.01$, *** $p<0.001$.

Supplementary Table 1 Primers used in this study

Gene	Forward	Reverse
For human		
GAPDH	GAAGATGGTGATGGGATTTC	GAAGGTGAAGGTCGGAGTC
ALB	CTGCCTGCCTGTTGCCAAAGC	GGCAAGGTCCGCCCTGTCATC
HNF4α	GGTGTCCATACGCATCCTTGAC	AGCCGCTTGATCTTCCCTGGAT
α1AT	TCGCTACAGCCTTTGCAATG	TTGAGGGTACGGAGGAGTTCC
FASN	TTCTACGGCTCCACGCTCTTCC	GAAGAGTCTTCGTCAGCCAGGA
UTX	AGCGCAAAGGAGCCGTGGAAAA	GTCGTTCACCATTAGGACCTGC
JMJD3	GACCCTCGAAATCCCATCACAG	GTGCGAACTTCCACGGTGTGTT
CYP3A4	TCTGTGCCTGAGAACACCAGAG	TGCATGTACAGAATCCCCGGTT
CYP2C9	AGGAAAAGCACAACCAACCATC	TTCAGCAGGAGAAGGAGAGCAT
CYP2C19	CTGGCTGAAAGAGCTAACAGAGG	TGAACACGGTCCTCAATGCTCC
ASS1	GCTGAAGGAACAAGGCTATGACG	GCCAGATGAACTCCTCCACAAAC
BSEP	AGCCACACAGACCAGGATGTTG	CAATGAACCGCCTCTCCTTTCC
MDR1	TTGCTGCTTACATTCAGGTTTCA	AGCCTATCTCCTGTCGCATTA
HADH	TCCGTTGTCCACAGCACAGACT	GGAGGAAGTGTTGCTGGCAAAG
CPT1A	GATCCTGGACAATACCTCGGAG	CTCCACAGCATCAAGAGACTGC
Chip-qPCR		

HADH	GGCCAAGCTGCTATCCAGT	ACGTTGGACGTCTATCAGTGC
For mouse		
GAPDH	TTGTCTCCTGCGACTTCAAC	GTCATACCAGGAAATGAGCTTG
ALB	CAGTGTTGTGCAGAGGCTGACA	GGAGCACTTCATTCTCTGACGG
CPS1	CATGGAACATCCAGCCGAATTGG	GATGGCACATCCTCAGAGCCTT
TTR	GAGTAGAACTGGACACCAAATCG	CTGCGATGGTGTAGTGGCGATG
ABCB1	TCCTCACCAAGCGACTCCGATA	ACTTGAGCAGCATCGTTGGCGA
HADH	TTCCAGAGGCTGGACAAGTTCG	GCCAGCAAATCGGTCTTGTCTG

Supplementary Table 2 Antibodies used in this study

Primary antibody				
Antibody	Company	Product number	Ig Species	Dilution
Anti-GAPDH	Proteintech	60004-1-Ig	Mouse	30000
Anti-ALB	Bethyl	A80-129A	Goat	1000
Anti-α1AT	Bethyl	A80-122A	Goat	200
Anti-MDR1	CST	13342	Rabbit	200
Anti-UTX	BOSTER	A01286-2	Rabbit	1000
Anti-JMJD3	Abcepta	AP1022A	Rabbit	1000
Anti-human/mouse H3	CST	4499T	Rabbit	1000
Anti-human/mouse H3K27me3	CST	9733T	Rabbit	1000

Anti-human/mouse H3K27ac	CST	8173T	Rabbit	1000
Anti-H3K18ac	CST	9675T	Rabbit	1000
Anti-H3K9ac	CST	9649T	Rabbit	1000
Anti-FASN	Abclonal	A19050	Rabbit	1000
Anti-CPT1A	Proteintech	15184-1-AP	Rabbit	1000
Anti-human/mouse HADH	Proteintech	19828-1-AP	Rabbit	1000
Anti-mouse ALB	Abcepta	AP6540B	Rabbit	1000
Anti-EP300	CST	86377	Rabbit	1000
Anti-Factin (Phalloidin-iFluor 594)	Abcam	ab176757		1000
Secondary antibody	Company	Product number	Dilution	
Anti-rabbit IgG, HRP-linked antibody	Beyotime	A0208	800	
Anti-mouse IgG, HRP-linked antibody	Beyotime	A0350	800	
Donkey anti-goat IgG, HRP-linked antibody	Beyotime	A0358	800	
Alexa Fluor 647-conjugated donkey anti-Goat IgG	Abcam	ab150135	800	

Supplementary Table 3 shRNA sequence used in this study

Gene	Sequence
UTX-1	CCGGTTTATTCCTTAGTCTATGTGCCTCGAGGCACATAGACTAAGGAATAAATTTTTG
UTX-2	CCGGGCAGCACGAATTAAGTATTTACTCGAGTAAATACTTAATTCGTGCTGCTTTTTG
JMJD3-1	CCGGCTCCTACACCCAGCATTTATTCTCGAGAATAAATGCTGGGTGTAGGAGTTTTTG
JMJD3-2	CCGGCTGTTCGTGACAAGTGAGACTCGAGTCTCACTTGTCACGAACAGTTTTTG
HADH-1	CCGGGATTATGTCGGACTGGATACTCTCGAGAGTATCCAGTCCGACATAATCTTTTTG
HADH-2	CCGGTCCAAACGTGTGGTGATTCTACTCGAGTAGAATCACCACACGTTTGGATTTTTG

Supplementary Table 4 Results of GO enrichment analysis corresponding to Fig. 4e

ID	Description	GeneRatio	pvalue	geneID
GO:0006805	xenobiotic metabolic process	8/140	2.77E-06	FMO1/CYP2C18/GSTM3/NR1I2/GSTO1/CYP2C19/SULT1A1/ACY1
GO:0017144	drug metabolic process	5/140	2.25E-05	FMO1/CYP2C18/NR1I2/CBR1/CYP2C19
GO:0009306	protein secretion	12/140	0.000173	CFTR/SPHK2/RAB3D/ADTRP/TGFB3/NR0B2/HADH/RCN3/PDIA4/SLC25A6/CAVIN1/HCAR2
GO:0035592	establishment of protein localization to extracellular region	12/140	0.000177	CFTR/SPHK2/RAB3D/ADTRP/TGFB3/NR0B2/HADH/RCN3/PDIA4/SLC25A6/CAVIN1/HCAR2
GO:0006631	fatty acid metabolic process	11/140	0.000183	TECR/CYP2C18/ADTRP/EHHADH/HADH/SLC27A2/CBR1/BDH2/CYP2C19/PLA2G4F/ECI2
GO:0006635	fatty acid beta-oxidation	5/140	0.000224	EHHADH/HADH/SLC27A2/BDH2/ECI2
GO:0098754	detoxification	6/140	0.000578	AKR1A1/TXNDC17/GSTM3/DUOX2/GSTO1/PDZK1
GO:0008202	steroid metabolic process	9/140	0.000846	CFTR/NR0B2/RDH5/SLC27A2/NR1I2/TTC39B/CYP2C19/SULT1A1/DHRS11
